# Engineering endogenous ABC transporter with improving ATP supply and membrane flexibility enhances the secretion of β-carotene in *Saccharomyces cerevisiae*

**DOI:** 10.1186/s13068-020-01809-6

**Published:** 2020-10-10

**Authors:** Xiao Bu, Jing-Yuan Lin, Jing Cheng, Dong Yang, Chang-Qing Duan, Mattheos Koffas, Guo-Liang Yan

**Affiliations:** 1 Centre for Viticulture and Enology, College of Food Science and Nutritional Engineering, China Agricultural University, 17 East Tsinghua Rd, Beijing, 100083 China; 2 Key Laboratory of Viticulture and Enology, Ministry of Agriculture and Rural Affairs, Beijing, 100083 China; 3 Beijing Key Laboratory of Functional Food From Plant Resources, College of Food Science and Nutritional Engineering, China Agricultural University, Beijing, 100083 China; 4 Center for Biotechnology and Interdisciplinary Studies and Department of Chemical and Biological Engineering, Rensselaer Polytechnic Institute, Troy, NY 12180 USA

**Keywords:** Endogenous ABC transporters, Biofuel products, ATP, Membrane fluidity, *Saccharomyces cerevisiae*

## Abstract

**Background:**

Product toxicity is one of the bottlenecks for microbial production of biofuels, and transporter-mediated biofuel secretion offers a promising strategy to solve this problem. As a robust microbial host for industrial-scale production of biofuels, *Saccharomyces cerevisiae* contains a powerful transport system to export a wide range of toxic compounds to sustain survival. The aim of this study is to improve the secretion and production of the hydrophobic product (β-carotene) by harnessing endogenous ABC transporters combined with physiological engineering in *S. cerevisiae*.

**Results:**

Substrate inducibility is a prominent characteristic of most endogenous transporters. Through comparative proteomic analysis and transcriptional confirmation, we identified five potential ABC transporters (Pdr5p, Pdr10p, Snq2p, Yor1p, and Yol075cp) for β-carotene efflux. The accumulation of β-carotene also affects cell physiology in various aspects, including energy metabolism, mitochondrial translation, lipid metabolism, ergosterol biosynthetic process, and cell wall synthesis. Here, we adopted an inducible GAL promoter to overexpress candidate transporters and enhanced the secretion and intracellular production of β-carotene, in which Snq2p showed the best performance (a 4.04-fold and a 1.33-fold increase compared with its parental strain YBX-01, respectively). To further promote efflux capacity, two strategies of increasing ATP supply and improving membrane fluidity were following adopted. A 5.80-fold increase of β-carotene secretion and a 1.71-fold increase of the intracellular β-carotene production were consequently achieved in the engineered strain YBX-20 compared with the parental strain YBX-01.

**Conclusions:**

Overall, our results showcase that engineering endogenous plasma membrane ABC transporters is a promising approach for hydrophobic product efflux in *S. cerevisiae*. We also highlight the importance of improving cell physiology to enhance the efficiency of ABC transporters, especially energy status and cell membrane properties.

## Introduction

Metabolic engineering and synthetic biology efforts have been input to produce various biofuels and chemicals in microbial hosts [1, 2]. However, the product toxicity introduces an undesirable metabolic burden, which subsequently limits titers and impacts process economics [3]. To address toxicity issue, several feasible strategies have been adopted, such as decoupling cell growth with production phases [4], modifying the components of the global transcription machinery [5], directed evolution [6], membrane engineering [7], and transport engineering [8–10]. Among them, transport engineering is considered to be one of the most promising strategies, because it not only relieving the product toxicity but also potentially reducing the separation costs [11, 12]. Furthermore, enhance the efflux of toxic products could avoid negative feedback regulation [9, 13].

Unlike isoprene (C5) which can be automatically released from cells and collected from the headspace of culture medium [14], most biofuel molecules are synthesized and stored inside cells, which requires membrane transporters to secret out of cells. ATP-binding cassette (ABC) transporters are known for their detoxification functions in prokaryotic and eukaryotic cells and have been demonstrated to export a diverse range of hydrophobic compounds [15]. As a model organism, engineering heterogonous prokaryotic ABC transporters to export biofuels have been systematically reported in *Escherichia coli* [8]. Several studies have reported efforts to enhance efflux using exogenous ABC transporters in yeast [11, 16]. For instance, Lee and co-workers transformed *S. cerevisiae* ABC transporter Pdr10p in *Rhodosporidium toruloides* enhanced the secretion of carotenoids [11]. Interestingly, there were reports demonstrating that overexpression of many heterologous efflux pumps was less effective in the efflux of biofuels as compared to the endogenous transporters [8, 9], which might be due to the poor expression/functional activities of these heterologous efflux pumps [10]. Therefore, self-expression of transporters is believed to be a promising strategy for stable expression in cell factories [12]. As a robust cell factory, *S. cerevisiae* contains 11 plasma membrane ABC transporters, which guarantee cells pumping thousands of substances, and empower a strong tolerance against harsh conditions and toxic compounds [17, 18]. Thus, it is reasonable to believe that ABC proteins can potentially serve as the functional transporters to secret biofuel out of cells, and engineering these endogenous transporters might be an effective way to improve biofuel production in *S. cerevisiae*. However, to our knowledge, no such studies have been reported yet.

It should be pointed out that once targeted ABC transporter is identified and overexpressed in *S. cerevisiae*, three challenges need to be addressed. Firstly, it is vital to maintain a balance between cell growth and transporter expression [19]. Separating the cell growth phase and transporter expression phase by using phase-dependent promoters is an ideal strategy to alleviate the metabolic burden caused by transporter overexpression. Secondly, ABC transporters are ATP-based transporters, which hydrolyzing ATP to drive transport [20]. Therefore, sufficient ATP has to be supplied to the ABC transporter overexpressed strains. Thirdly, the overproduction of membrane proteins embedded in the cell membrane would increase the rigidity of the cell membrane and disturb its normal function, one consequence is decreasing cell growth and the transport capacity [21]. Maintaining the normal function of the cell membrane is therefore necessary to efficient export products, such as improving membrane fluidity [22].

**Fig. 1:**
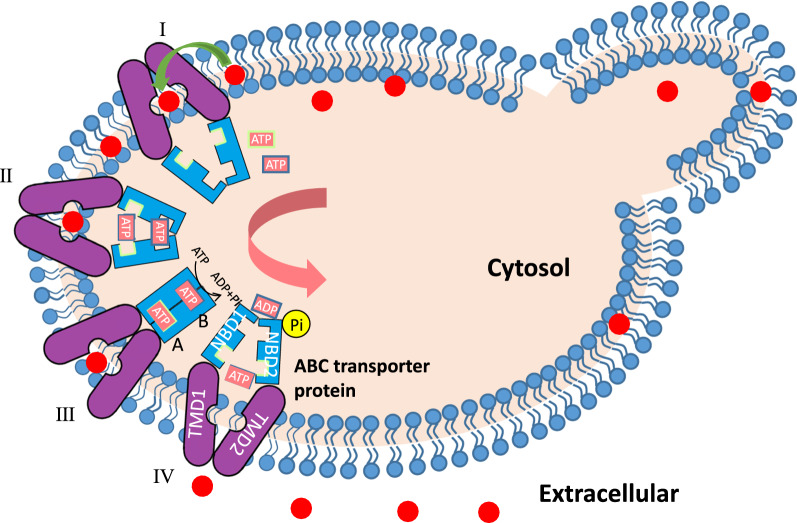
The secretion process of β-carotene by ABC transporters in *S. cerevisiae*. ABC transporters consist of two cytoplasmic nucleotide-binding domains (NBDs, blue) that hydrolyze ATP to drive transport and two transmembrane domains (TMDs, purple) that bind chemical compounds and provide a translocation channel. The specific secretion process of β-carotene is as follows: (I) β-carotene (red circles) is transferred from the cell membrane to the hydrophobic transmembrane domain when the transporter opens inward, (II) then ATP is bound at both sites. (III) ATP binding causes the dimerization of NBDs. The ATP hydrolysis is limited to the canonical one (step III-B), which drives a change from an inward-facing, drug-binding conformation to an outward-facing, drug-releasing conformation. (IV) When β-carotene is secreted out of the cell, the protein was reset in a drug-binding conformation

Potential biofuel compounds include derivatives of fatty acids, alcohols, isoprenoids, and polyketides [23]. In the study reported here, we engineered *S. cerevisiae* to enhance the secretion and production of β-carotene by harnessing its endogenous ABC transporters (Fig. 1, [24]). β-Carotene, as a type of isoprenoids, was chosen as a model biofuel compound for two main reasons: first, it has physical properties that more closely resemble petroleum-derived fuels, which is an attractive alternative for the future supplementation or replacement of petroleum-derived fuels [25]; second, it is readily detectable using colorimetric methods [8]. Through the proteomic analysis and transcriptional confirmation, we identified five potential plasma membrane ABC transporters for β-carotene efflux, which are up-regulated when β-carotene accumulate in cells. We then applied three strategies to improve β-carotene secretion: (i) control the expression level of ABC transporter by inducible GAL promoter; (ii) enhance intracellular ATP supply; (iii) improve cell membrane fluidity. With these efforts, a 5.80-fold increase of β-carotene secretion and a 1.71-fold increase of intracellular β-carotene production were observed compared with the parental strain YBX-01. The strategies in this study provide new insights into the use of endogenous ABC transporters to enhance biofuels production in *S. cerevisiae*.

## Results and discussion

### Mining potential transporters for β-carotene efflux

**Fig. 2:**
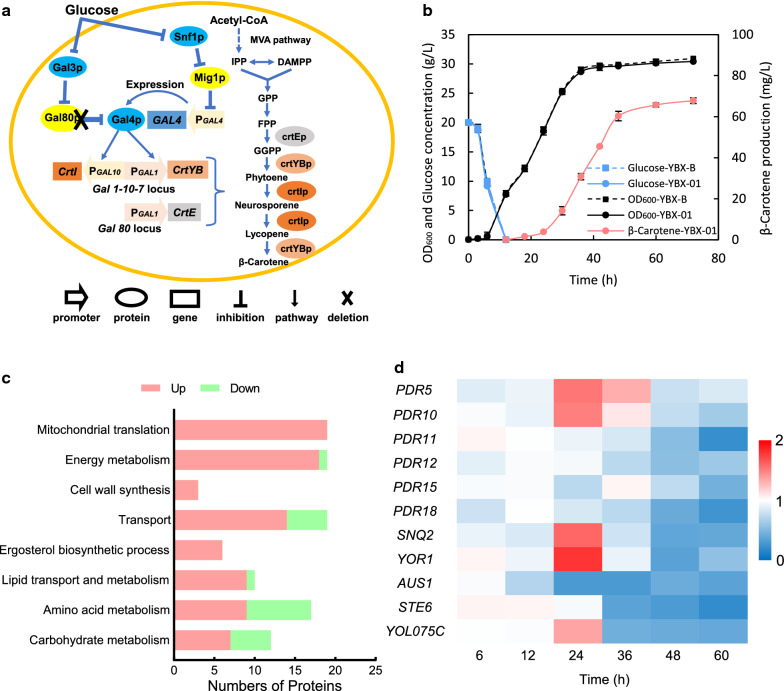
β-Carotene biosynthesis in *S. cerevisiae* and cell response to β-carotene accumulation. **a** Schematic overview of the β-carotene biosynthetic pathway in *S. cerevisiae*. The expression of galactose utilization related genes is mainly controlled by proteins Gal4, Gal3 and Gal80. Gal4 is a transcriptional regulatory protein, which can specifically bind to the promoters of the *GAL1*, *GAL7*, *GAL10*, and *GAL2* genes to activate the transcription of the aforementioned genes. Gal80 is the inhibitory protein of Gal4. When yeast cells grow in a culture without galactose, Gal80 can specifically bind to Gal4, preventing the binding of Gal4 protein to the promoter region of *GAL* genes, thereby inhibiting the expression of *GAL* genes. When the gene *GAL80* is knocked out, the expression of *GAL4* is only controlled by the Snf1 regulatory network. After the glucose in the medium is exhausted, the inhibition of *GAL4* is relieved, then the *GAL* genes begin to express and drive the product synthesis. The dashed lines indicate multiple step reactions. *MVA pathway* mevalonate pathway, *IPP* isopentenyl pyrophosphate, *DMAPP* dimethylallyl pyrophosphate, *GPP* geranyl pyrophosphate, *FPP* farnesyl pyrophosphate, *GGPP* geranylgeranyl pyrophosphate, *crtEp* GGPP synthase, *crtYBp* encoding bifunctional phytoene synthase and lycopene cyclase, *crtIp* phytoene desaturase. **b** Time courses of cell growth, the glucose concentration of β-carotene producing strain YBX-01 and its parental strain YBX-B and β-carotene accumulation of YBX-01. **c** The comparative proteome analysis between YBX-01 and YBX-B at 36 h. **d** The comparative transcription levels of plasma membrane ABC transporters between YBX-01 and YBX-B throughout the cell growth period. All data represent the mean ± s.d. of biological triplicates

Substrate inducibility is a prominent characteristic of most endogenous transporters [26]. Thus, omics analysis under product-induced stress could be used to identify the candidate transporters [27]. In this study, to reduce the toxicity of carotenoid on cell growth, we adopted an inducible GAL promoter to drive the β-carotene biosynthesis (Fig. 2a), which can effectively separate the cell growth stage from the product accumulation stage [28]. As illustrated in Fig. 2b, there was no significant difference in cell growth and glucose consumption between the β-carotene producing strain YBX-01 and its parental strain YBX-B. After glucose was exhausted (12 h), the β-carotene started to synthesize and reached the highest intracellular concentration of 67.8 mg/L of β-carotene after 72 h of cultivation.

To discover potential transporters for β-carotene efflux, we integrated comparative proteomic analysis and transcriptional confirmation between YBX-01 and YBX-B. Firstly, comparative proteomics was carried out using iTRAQ-based LC–MS/MS analysis after 36 h of fermentation, which corresponds to the time point of highest β-carotene synthesis rate achieved (Additional file 1: Fig. S1). A total of 105 proteins showed different expression patterns (at least fold change ≥ 1.2, *P* < 0.05, Additional file 1: Table S1). Compared with the reference strain, 85 proteins were up-regulated and 20 proteins were down-regulated in YBX-01. Specifically, the amount of Pdr5p (1.20-fold) and Pdr10p (1.26-fold) involved in the multi-drug resistance network of yeast were increased in YBX-01. This is corresponding to previous results that *PDR5* and *PDR10* were induced in recombinant yeast to export heterologous carotenoids to reduce product toxicity [29]. The other 12 up-regulated transporters were involved in the transport of substances such as protons, metal ions or salt ions (Pma2p, Ady2p, Smf1p, Alr1p and Fre1p), amino acids (Mup1p) and sugars (Gal2p), or involve the transport of substances inside the cell (Odc1p, Yhm2p, Erp3p, Chs6p and Pxa1p). Besides the transport processes, we also observed significant changes in protein expression levels related to energy metabolism, mitochondrial translation, lipid metabolism, ergosterol biosynthetic process, and cell wall synthesis (Fig. 2c). For example, the mitochondrial proteins involved in TCA cycle (Sdh1p, Sdh2p Sdh3p, Lsc1p, Lsc2p, Cit2p and Cit3p) and ATP synthesis (Atp7p, Atp17p, Atp19p, Atp20p and Tim11p) were up-regulated. This indicated that the ATP generation system became abnormal due to the β-carotene accumulation, which might lead to ATP deficiency in the YBX-01 strain, as shown in our previous study [30]. The proteins related to lipid metabolism (such as Acc1p, Ino1p and Cyb5p) and ergosterol biosynthetic process (Erg1p, Erg4p, Erg6p, Erg20p, Erg24p, and Erg25p) were simultaneously enriched in YBX-01 strain (Additional file 1: Table S1). This confirmed the occurrence of membrane stress in recombinant yeast and the necessity of improving membrane flexibility for efficient synthesizing carotenoid [22, 31]. We also observed the amounts of cell wall synthesis proteins (Mpg1p, Crh1p, and Pst1p) raised in YBX-01. Collectively, the data of comparative proteomics suggested that despite the minimal impact on cell growth due to the application of an inducible GAL promoter to drive the β-carotene biosynthesis, the accumulation of β-carotene still significantly affected protein expression in various aspects. As a response, cells activate a series of defense mechanisms to tackle these stresses.

With the help of proteomic analysis, we identified two potential ABC transporters for β-carotene efflux: Pdr5p and Pdr10p. There are two main reasons for the decision to consider ABC family as candidate transporters. Firstly, ABC proteins of yeast are implicated in the transport of lipids or hydrophobic molecules [15]. Secondly, other multi-drug resistance transporter family in yeast do not respond to β-carotene accumulation (Additional file 1: Table S1). Considering that there are 11 ABC plasma membrane transporters in *S. cerevisiae*, their expression varies with cell physiology and growth period [18], measuring proteomics profile at a single time point could probably miss some valuable transporters. To assess expression profiling changes of ABC transporters among the different time-points, we compared the transcriptional profiles of all 11 ABC transporters between YBX-01 and YBX-B throughout the cell growth period (including 6 h, 12 h, 24 h, 36 h, 48 h and 60 h, Fig. 2d) by qPCR analysis. In the early log phase (6 h and 12 h), there was no significant difference in the expression levels of all ABC transporters between the two strains, since the biosynthesis of carotenoid has not been initiated (Fig. 2b). As β-carotene began to accumulate intracellularly in YBX-01 after 24 h of cultivation, five ABC transporters were distinctly induced compared with YBX-B, including *PDR5* (1.54-fold), *PDR10* (1.50-fold), *SNQ2* (1.61-fold), *YOR1* (1.79-fold), and *YOL075C* (1.36-fold). In contrast, the expression levels of the additional six transporter genes (*PDR11*, *PDR12*, *PDR15*, *PDR18*, *AUS1* and *STE6*) did not increase. We hypothesized that they may not participate or have a limited effect on β-carotene transportation. It is worth noting that with the continuous accumulation of β-carotene, the expression level of all ABC transporters in YBX-01 did not increase further, conversely, showed decreased profiles compared with YBX-B. We speculated that the excessive accumulation of β-carotene on the cell membrane might interfere with the expression of transporters [32]. Herein, via the integration of proteomic analysis and transcriptional confirmation, we systematically understanding the physiological response of cells to β-carotene accumulation and identified five potential ABC transporters (including Pdr5p, Pdr10p, Snq2p, Yor1p and Yol075cp) for β-carotene efflux.

### Individual overexpression of ABC transporters facilitated β-carotene efflux

Here, we individually overexpressed the five ABC transporters in the YBX-01 strain. The additional six ABC transporters were also individually overexpressed to verify the effectiveness of the method of tapping potential transporters (Additional file 1: Fig. S2). To alleviate the cytotoxicity of transporter overexpression, we replaced the natural promoter of ABC transporter genes with the strong inducible *GAL1* promoter (Additional file 1: Fig. S3), which achieved the synchronous synthesis of transporters and products. A series of plasmids were constructed that can replace the promoter of the target genes (Additional file 1: Table S2). Transformants were confirmed by genomic PCR (Additional file 1: Table S3, Fig. S4), and qPCR analysis confirmed increased expression levels of all the target genes (ranging from 25- to 733-fold) over those of the wild-type controls (Additional file 1: Fig. S5).

**Fig. 3:**
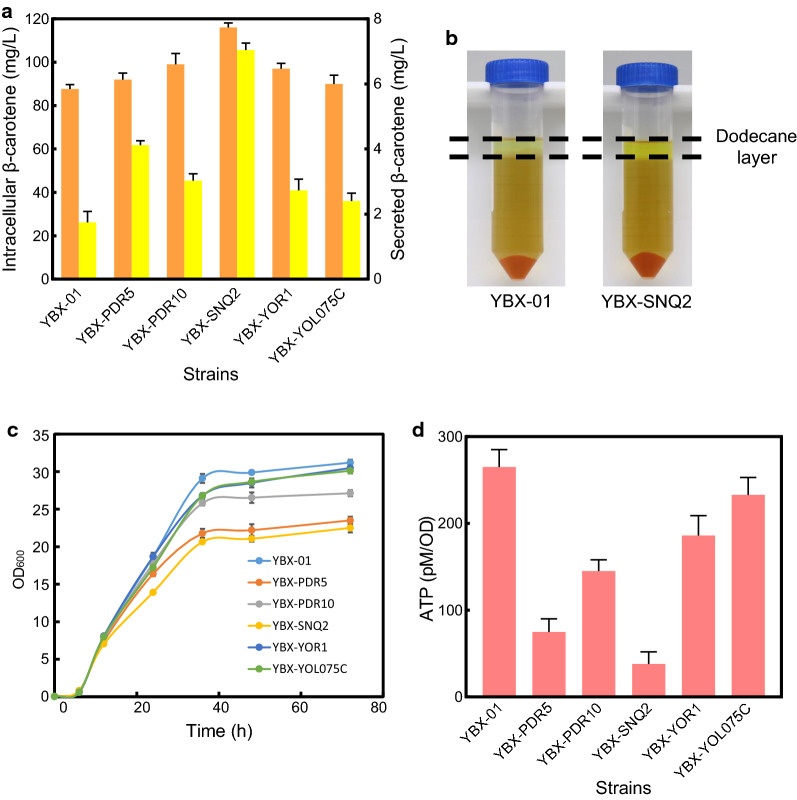
The secretion of β-carotene in ABC transporter overexpression strains. **a** The intracellular (in orange) and secreted (in yellow) β-carotene in ABC transporter overexpression strains. **b** After the centrifugation, the β-carotene in the dodecane layer can be seen in the top, the YPD medium in the middle, and the cell pellet at the bottom. **c** Cell growth of ABC transporter overexpression strains. **d** Intracellular ATP content of ABC transporter overexpression strains. All data represent the mean ± s.d. of biological triplicates

Two-phase fermentation system with dodecane as an extractive solvent was employed to circumvent the insolubility issue [33]. The addition of dodecane (10% [v/v]) in YBX-01 did not deter cell growth and glucose consumption, and unexpectedly improve the cell growth slightly (Additional file 1: Fig. S6). With dodecane addition, 1.74 mg/L β-carotene was secreted into the dodecane layer after 72 h of cultivation in YBX-01 (Fig. 3a). In terms of the dynamic changes in β-carotene efflux level throughout the cell growth period, we found that with the extension of culture time, β-carotene efflux level in YBX-01 increased continuously, which was consistent with gene expression of the five transporters (Additional file 1: Fig. S7). It is worth noting that the intracellular β-carotene production was also increased from 67.8 to 87.7 mg/L. There are three reasons for this phenomenon. Firstly, the oxygen dissolved better in dodecane, and more oxygen in the medium is beneficial to carotenoid biosynthesis due to surplus NADH can be re-oxidized through respiratory metabolism pathways [34]. Secondly, the secretion of β-carotene from cells reduces its cytotoxicity and increases cell tolerance, which facilitates intracellular β-carotene production [9]. Thirdly, dodecane as a carbon source may promote the β-carotene biosynthesis.

Compared to YBX-01, individual overexpression of the five ABC transporters increased the secretion level of β-carotene (Fig. 3a). In particular, YBX-SNQ2 showed the highest β-carotene secretion level (7.04 mg/L, Fig. 3b), with 4.04-fold higher than that of the parental strain YBX-01, followed by YBX-PDR5 (2.37-fold), and YBX-PDR10 (1.74-fold). Snq2p is functionally homologous to Pdr5p, and was identified as a protein conferring resistance to 4NQO (4-nitroquinoline 1-oxide). Overexpression of *SNQ2* confers resistance to anticancer drugs, antibiotics, fungicides and many other chemicals [35]. As reported by Lee and co-workers, *S. cerevisiae* Pdr10p engineered into the oleaginous yeast *R. toruloides* led to a sixfold increase of carotenoid secretion [11]. However, in this study, only a 1.74-fold increase was observed in YBX-PDR10, indicating that the background of host has a significant effect on the genetically modified phenotype [36]. Our results demonstrated that multiple ABC transporters in *S. cerevisiae* are involved in β-carotene efflux, and their combined overexpression might have a synergistic effect. Therefore, two transporters Snq2p and Pdr5p, which have a strong ability to export β-carotene, were co-overexpressed in YBX-01. However, co-overexpression of the two transporters did not synergistically promote β-carotene efflux and production (data not shown), possibly due to the overexpressed membrane proteins that cause membrane overload and thus interfere with product transportation [19]. Precisely controlling the expression levels of transporter genes or using a feedback regulation system might be an effective way to avoid the membrane overload and achieve increased β-carotene efflux [13, 19].

As illustrated in Fig. 3a, compared with the control strain, all five ABC transporter-overexpressing strains have higher intracellular β-carotene content, confirming that increasing the efflux capacity is beneficial to biofuel production [10]. Correspondingly, the highest intracellular level of β-carotene was found in YBX-SNQ2 (116.3 mg/L), which was 1.32-fold higher than that of parental strain YBX-01. We also tested the secretion phenotypes of the additional six ABC transporters and found that overexpression of these transporters did not improve the secretion and production of β-carotene significantly (Additional file 1: Fig. S2), which indicates that the transcriptional analysis is a powerful tool to discover the endogenous transporters [37].

Although using an inducible GAL promoter, overexpression of efflux pumps still inhibited cell growth to varying degrees (Fig. 3c). Especially the overexpression of Pdr5p and Snq2p, the final biomass (OD_600_) of the two strains (YBX-PDR5 and YBX-SNQ2) decreased by 24.7% and 27.9%, respectively. Interestingly, the overexpression of Yor1p and Yol075cp have no obvious inhibition effect on cell growth, indicated that excessive production of ABC transporters may not the main cause for growth arrest. Considering that ABC transporters are ATP-driven proteins, we speculated that ATP depletion may be the main cause of biomass reduction.

To prove this hypothesis, the intracellular ATP content of various ABC transporter overexpressing strains were tested after cultivating for 36 h. As shown in Fig. 3d, intracellular ATP levels showed considerable variation among different strains. Specifically, the ATP content in YBX-PDR5 and YBX-SNQ2 were dramatically decreased by 71.7% and 85.7% relative to the control strain YBX-01, respectively. In comparison, only 29.8% and 12.1% decrement were observed in YBX-YOR1 and YBX-YOL075C, respectively. The correlation between the reduction in energy and the decrease in biomass strongly supports our hypothesis that ATP depletion is the main cause of the decreased biomass. Therefore, to improve the secretion of β-carotene efficiently, it is vital to provide transporter overexpressing strains with additional ATP.

### Evaluation of ATP supply strategies

To increase product secretion efficiency, we developed three strategies to improve ATP supply, including exogenous addition of adenosine triphosphate (ATP) disodium, overexpressing mitochondrial genes related to ATP generation and acetate supplementation. The effectiveness of these strategies was first evaluated in the strain YBX-SNQ2 by determining the variation in β-carotene secretion levels.

**Fig. 4:**
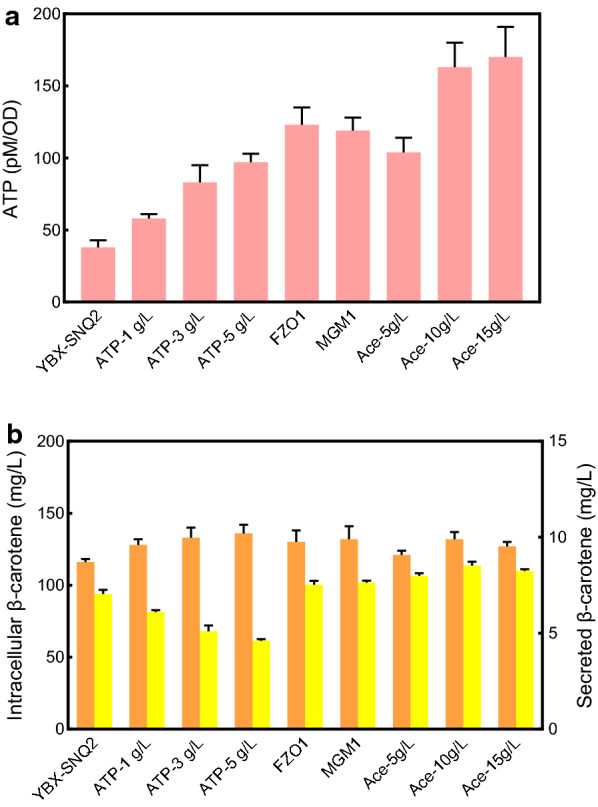
Different ATP supply strategies applied to YBX-SNQ2. **a** The intracellular ATP content of YBX-SNQ2 under different ATP supply strategies. **b** The intracellular (in orange) and secreted (in yellow) β-carotene of YBX-SNQ2 under different ATP supply strategies. All data represent the mean ± s.d. of biological triplicates

Exogenous addition of ATP in the form of ATP disodium salt has been shown to increase the production of glutathione in *Candida utilis* and *E. coli* by enhancing intracellular ATP levels [38, 39]. Therefore, we expected to increase intracellular ATP levels in the same way to promote β-carotene secretion. When β-carotene biosynthesis began (12 h), a series of concentrations of ATP disodium salt (1 g/L, 3 g/L, and 5 g/L) were added. As expected, the intracellular ATP raised correspondingly compared to the cultivation without ATP salt addition (Fig. 4a), which was paralleled with the increase of intracellular β-carotene production (Fig. 4b). However, the increased secretion of β-carotene was not observed, conversely, it decreased with the enhancement of ATP level. We speculated that this might be due to the repression of ATPase activity of ABC transporters by ATP disodium salt, owing to that ATPase activity of ABC transporters can be inhibited by salt (e.g., vanadate) [40].

The enrichment of mitochondrial proteins implied that the normal mitochondria function is disturbed by the accumulation of β-carotene (Fig. 1c). The rise of intracellular ROS content in YBX-01 also confirmed this conclusion (Additional file 1: Fig. S8). As a highly dynamic organelle, mitochondria constantly fuse and divide to maintain normal cellular functions and provide energy from oxidative phosphorylation. Overexpression of *FZO1* or *MGM1* promotes mitochondrial fusion, which helps maintain the integrity of mitochondrial DNA and thereby increasing the level of ATP supply [41]. Here, we overexpressed the yeast mitochondrial fusion genes *FZO1* and *MGM1* individually using the *GAL1* promoter. The results showed that *FZO1* overexpression increased the intracellular ATP content by 3.24-fold compared with the control strain YBX-SNQ2, which consequently raised intracellular (1.12-fold) and extracellular (1.07-fold) β-carotene content, respectively (Fig. 4). A similar phenomenon was observed in *MGM1* overexpression strain, with a 1.14-fold increase of intracellular and a 1.08-fold increase of extracellular β-carotene, respectively.

Acetate, an abundant and renewable resource, can improve protein secretion by increasing ATP content in *Schizosaccharomyces pombe* [42]. As a precursor of acetyl-CoA, acetate supplementation leads to an increase in carbon flux of the TCA cycle and promotes the synthesis of ATP [30]. In this study, different concentrations of acetate (5 g/L, 10 g/L, and 15 g/L) was added after 12 h cultivation of YBX-SNQ2, and 10 g/L acetate addition showed the best performance for β-carotene production and efflux. A 4.29-fold increase of intracellular ATP level was obtained in 10 g/L acetate addition relative to that without addition (Fig. 4a). For β-carotene production, 1.14-fold and 1.21-fold increase of intracellular (132.52 mg/L) and extracellular (8.52 mg/L) of β-carotene were achieved in 10 g/L acetate addition compared to the control, respectively (Fig. 4b). We further evaluated the effect of mitochondrial fusion gene (*FZO1* or *MGM1*) overexpression combined with acetate addition on β-carotene secretion. Unexpectedly, the combination strategy did not achieve the synergistic effect (data not shown).

In terms of cell growth (Additional file 1: Fig. S9), the addition of ATP salt slightly promoted the cell growth, while the overexpression of *FZO1* or *MGM1* had no significant effect on cell growth. Although the addition of acetate inhibited the cell growth of YBX-SNQ2 at the beginning, there were no significant differences in the final biomass between with and without acetate addition. Finally, considering its highest ability of improving β-carotene secretion level, 10 g/L acetate addition was selected as the best ATP enhancement strategy to improve the secretion of β-carotene in ABC transporter overexpression strains.

### Acetate supplementation enhances the β-carotene secretion in ABC transporter overexpression strains

**Fig. 5:**
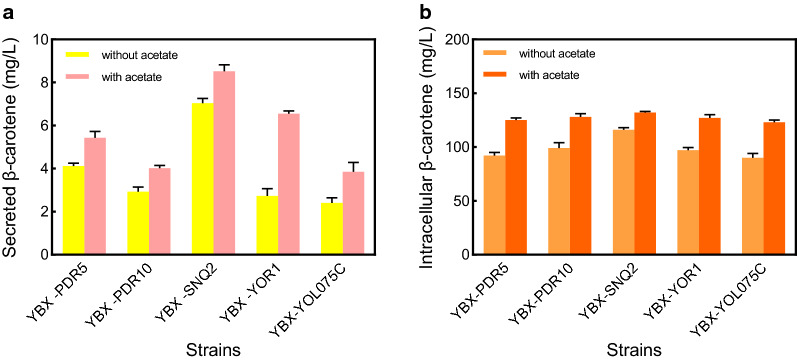
Acetate supplementation enhanced **a** secreted and **b** intracellular β-carotene levels in ABC transporter overexpression strains. All data represent the mean ± s.d. of biological triplicates

Considering that different transporters have different ATP utilization abilities, other ABC transporter overexpression strains may secrete more β-carotene than YBX-SNQ2 after additional ATP supplementation. Therefore, we tested the effect of 10 g/L acetate addition on β-carotene efflux of these ABC transporter overexpression strains. As shown in Fig. 5a, compared to the absence of acetate, β-carotene secretion levels were increased to varying degrees in the five engineered strains. The highest secreted amount was still found in YBX-SNQ2 (8.52 mg/L), which was 1.2-fold higher than the absence of acetate. It is noteworthy that the β-carotene secretion level in YBX-YOR1, which showed the relatively low value in the absence of acetate (2.73 mg/L), was increased by 2.4-fold and reached 6.56 mg/L. This suggests that the Yor1p is largely dependent on ATP for β-carotene efflux compare to other ABC transporters, which is consistent with the findings of Decottignies et al. that raising the ATP level in Yor1p-expressing yeast by supply glucose can significantly increase rhodamine B secretion [40].

Particularly necessary to point out that different from other transporters, ABC transporter contains two domains: the transmembrane domains (TMDs) that bind compounds and provide translocation channels, and the cytoplasmic nucleotide-binding domains (NBDs) that hydrolyze ATP for driving transport. The structure of TMDs and NBDs determine the specificity and efficiency of ABC transporter, which can be redesigned to improve the substrate specificity and transport activity, respectively. From the perspective of cellular economics, the energy-independent or less energy-dependent transporter may be the desired protein for product efflux [43]. In this sense, Snq2p may be the most suitable endogenous transporter for modification in future work because it can secret more β-carotene when consuming the same amount of ATP.

The intracellular β-carotene yield of the five strains were all significantly improved by acetate supplementation (Fig. 5b), and YBX-SNQ2 still showed the highest β-carotene production. Taken together, our results demonstrate that adding acetate can improve intracellular ATP content in yeast, thereby promoting the secretion of β-carotene in ABC transporter-overexpressing strains. The strain YBX-SNQ2 showed the best level of β-carotene intracellular production and secretion, which was therefore chosen as a target strain in the next step of modulating membrane flexibility.

### Improving membrane fluidity to enhance β-carotene secretion

Overproduction of efflux pumps or heterogenous biofuels anchor in the cell membrane can disorganize its structural integrity that impairs vital functions [16]. There are several works demonstrating that improving membrane fluidity by enriching intracellular unsaturated fatty acids (UFAs) can decrease the membrane stress [22, 31, 44]. Considering that membrane function greatly affects the secretion efficiency of products, we hypothesized that improving the membrane fluidity of host cells may be beneficial to β-carotene secretion. The *OLE1* gene encoding stearoyl-CoA 9-desaturase catalyzes the transformation of saturated fatty acids (C16:0 and C18:0) to UFAs (C16:1 and C18:1) through a dehydrogenation reaction. Overexpression of *OLE1* could improve the fatty acid unsaturation and membrane flexibility, which empower the cells with high tolerance to various types of stress [44–46]. In the present work, we tested the feasibility of improving cell membrane fluidity by overexpressing *OLE1* to enhance β-carotene secretion.

**Fig. 6:**
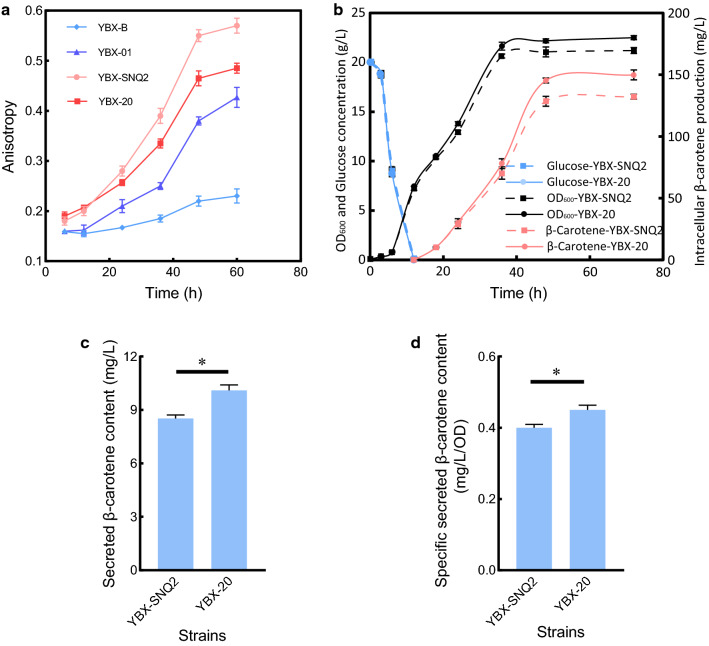
*OLE1* overexpression enhances the secretion level of β-carotene. **a** Anisotropy of strain YBX-B, YBX-01, YBX-SNQ2 and YBX-20. **b** Time courses of cell growth, glucose concentration and intracellular β-carotene production of YBX-SNQ2 and YBX-20. **c** The yield of secreted β-carotene in YBX-SNQ2 and YBX-20. **d** The specifically secreted β-carotene in YBX-SNQ2 and YBX-20. All data represent the mean ± s.d. of biological triplicates. *P < 0.05

By replacing the natural promoter with the *TEF1* promoter, *OLE1* was overexpressed in YBX-SNQ2 to obtain strain YBX-20. The membrane fluidity of YBX-B, YBX-01, YBX-SNQ2 and YBX-20 was determined throughout the cell growth phase. As shown in Fig. 6a, with the extension of cultivation time, the cell membrane fluidity of the strain YBX-B decreased slightly, indicated by the increment of anisotropy values. While the strains YBX-01, YBX-SNQ2 and YBX-20 exhibited sharp decreases in membrane fluidity, which is partially caused by the overload of β-carotene and transport proteins on cell membranes. As expected, overexpression of *OLE1* significantly abrogated the rate of decline in membrane fluidity in YBX-20 than in YBX-SNQ2. The fluorescence anisotropy of YBX-20 was 85% of YBX-SNQ2 at 60 h, demonstrated the effectiveness of *OLE1* in promoting cell membrane fluidity.

In the presence of acetate supplementation, *OLE1* overexpression resulted in the slight improvement of biomass (from 21.2 to 22.5 [OD_600_]) and intracellular β-carotene production (from 132.5 to 149.8 mg/L) simultaneously (Fig. 6b), probably due to the alleviation of membrane stress and decrement of product toxicity on the host strain [22]. More importantly, 18.8% increased secretion of β-carotene (10.1 mg/L) was observed in the YBX-20 strain compared with the strain YBX-SNQ2 (Fig. 6c). Due to the simultaneous cell growth improvement, we calculated the specific secretion coefficient of β-carotene in both strains to verify whether the increased β-carotene secretion results from improved biomass. The specific yield of secreted β-carotene 0.45 mg/L/OD in YBX-20 vs 0.40 mg/L/OD in YBX-SNQ2 (Fig. 6d) verified that the improvement of cell membrane fluidity promotes the secretion of β-carotene. As a widely applicable strategy, it is expected to be adopted in other microbial hosts to facilitate biofuels secretion.

## Conclusion

In this work, we aim to harness the endogenous ABC transporters to increase the secretion and production of hydrophobic compound (β-carotene) in *S. cerevisiae*. Through proteomic analysis and transcriptional confirmation, we systematically understood the response of cells to β-carotene accumulation and identified five potential ABC transporters for β-carotene efflux, including Pdr5p, Pdr10p, Snq2p, Yor1p and Yol075cp. Individual overexpression of the five ABC transporters indicated that Snq2p is the best endogenous transporter for β-carotene efflux. To efficiently improve product efflux, increasing ATP supply and improving membrane flexibility were implemented along with the ABC transporter overexpression. In general, the secretion and intracellular β-carotene production of strain YBX-20 were 10.1 mg/L and 149.8 mg/L respectively, which were 5.80-fold and 1.71-fold higher than that of the parental strain YBX-01. Our study demonstrates that harnessing endogenous transporters of *S. cerevisiae* can increase the efflux and production of biofuels, and highlights the importance of improving cell physiology to enhance the efficiency of transporters, especially energy status and cell membrane properties.

## Methods

### Strains, media and reagents

*E. coli* DH5α (Tiangen biotech, Beijing, China) was used for gene cloning. The yeast strain *S. cerevisiae* FY1679-01B (*MATα*; *ura3-52*) was used as the host for DNA integration and β-carotene production in this study. LB (Luria–Bertani broth) medium with antibiotics (50 mg/mL of kanamycin) was used for the cultivation of recombinant *E. coli*. Yeast extract–peptone–dextrose (YPD) medium consisting of 1% yeast extract, 2% peptone and 2% dextrose was used to cultivate yeast strains for competent cell preparation and shake flask fermentation. YPD medium containing 200 mg/mL geneticin was used for selection of the *KanMX* marker. SD-FOA (SD complete medium with 1 mg/mL 5-fluoroorotic acid) was used for selection of yeast strains with *KanMX-URA3-PRB322ori* marker excision. All restriction enzymes were purchased from Takara (Dalian, China). The standard β-carotene, antibiotics and chemicals were purchased from Sigma (Sigma Aldrich, USA).

### Plasmid construction

All plasmids used in this study were listed in Additional file 1: Table S2. All primers (Additional file 1: Table S3) were ordered from Sangon Biotech (Shanghai, China). DNA fragments, promoters, and homologous arms were PCR amplified from the genomic DNA of *S. cerevisiae* FY1679-01B. Plasmid pUMRI-21 kindly provided by prof. Hong-wei Yu [14] was used as a template for DNA construction in the yeast genome. Plasmid DNA and PCR products were purified using HiPure Plasmid Plus Maxi Kit and HiPure PCR Pure Maxi Kit (Magen, Guangzhou, China). The In-Fusion HD Cloning kits were purchased from Vazyme (Nanjing, China). For the detailed construction procedure of the promoter replacement plasmids, please see Additional file 1: Additional methods section—*Promoter replacement plasmids construction*.

### Yeast strain construction

The *S. cerevisiae* strains used in this work are listed in Table 1. pUMRI derivative plasmids were linearized from the junction of homologous arms with corresponding restriction enzymes and integrated into yeast genome by the lithium acetate/polyethylene glycol/single-stranded carrier DNA transformation method [47]. Recombinant strains were selected by G418 or uracil plates. All transformants were further evaluated by genomic DNA PCR using verification primers (Additional file 1: Table S3). After PCR confirmation, all the correct colonies were passaged overnight at 30 °C, 220 rpm. Subsequent recombination between the duplicated *loxp* flanks results in 5-FOA resistance due to *URA3* excision [48]. 5-FOA-resistant colonies were picked and checked for loss of the targeted marker by replica-plating on YPD and YPD–G418 plates. The promoter replacement schematic in yeast genome is shown in Additional file 1: Fig. S3.

**Table 1:** Yeast strains used in this study

Yeast strain	Parental strain	Genotype^a^	Source
FY1679-01B	S288C	*MATa*; *ura3-52*	Euroscarf
YBX-YB/I	FY1679-01B	*Δgal1-10-7::T*_*ADH1*_*-CrtI-P*_*GAL10*_*-P*_*GAL1*_*-CrtYB-T*_*CYC1*_	This study
YBX-01	YBX-YB/I	*Δgal80::T*_*ADH1*_*-CrtE-P*_*GAL10*_	This study
YBX-B	FY1679-01B	*Δgal1-10-7, Δgal80*	This study
YBX-PDR5	YBX-01	*ΔP*_*PDR5*_*::P*_*GAL1*_	This study
YBX-PDR10	YBX-01	*ΔP*_*PDR10*_*::P*_*GAL1*_	This study
YBX-PDR11	YBX-01	*ΔP*_*PDR11*_*::P*_*GAL1*_	This study
YBX-PDR12	YBX-01	*ΔP*_*PDR12*_*::P*_*GAL1*_	This study
YBX-PDR15	YBX-01	*ΔP*_*PDR15*_*::P*_*GAL1*_	This study
YBX-PDR18	YBX-01	*ΔP*_*PDR18*_*::P*_*GAL1*_	This study
YBX-SNQ2	YBX-01	*ΔP*_*SNQ2*_*::P*_*GAL1*_	This study
YBX-YOR1	YBX-01	*ΔP*_*YOR1*_*::P*_*GAL1*_	This study
YBX-AUS1	YBX-01	*ΔP*_*AUS1*_*::P*_*GAL1*_	This study
YBX-STE6	YBX-01	*ΔP*_*STE6*_*::P*_*GAL1*_	This study
YBX-YOL075C	YBX-01	*ΔP*_*YOL075C*_*::P*_*GAL1*_	This study
YBX-FZO1	YBX-01	*ΔP*_*FZO1*_*::P*_*GAL1*_	This study
YBX-MGM1	YBX-01	*ΔP*_*MGM1*_*::P*_*GAL1*_	This study
YBX-20	YBX-SNQ2	*ΔP*_*OLE1*_*::P*_*TEF1*_	This study

^a^The description of the genotype in the table is based on the parental strain

### Cultivation in shaking flask

For shake flask culture, fresh single colony was picked out from the plate and inoculated into 5 mL of YPD medium for overnight growth at 30 °C, 220 rpm in dark conditions. The resulting seed cultures were used to inoculate 250-mL flasks containing 50 mL YPD medium to an initial OD_600_ of 0.05 and grown under the same condition for 72 h. Cell growth was monitored by measuring OD_600_ on a spectrophotometer. After 72 h of growth, the cells were collected to measure the β-carotene production. Dodecane was added to the culture at a volumetric ratio of 1:10 to form a hydrophobic phase above the culture phase. The extracellular β-carotene was extracted from the supernatant by using dodecane, owing to its high hydrophobicity (log *Pow* 7.0) and insignificant toxicity toward *S. cerevisiae* [37].

### Analysis and quantification of carotenoid in intracellular and organic phase

The intracellular carotenoid was extracted using hot HCl-acetone [28]. The analyses of β-carotene were performed on a HPLC system (Agilent 1200 LC) equipped with a C18 column (4.6 mm × 150 mm) and the UV/VIS signals were detected at 450 nm. The mobile phase consisted of acetonitrile–methanol–isopropanol (50:30:20 v/v) with a flow rate of 1 mL/min at 40 °C.

To determine the maximum absorption wavelength of β-carotene standard in dodecane, wavelength scan from 400 to 500 nm was performed, the λ_max_ is 455 nm. The standard curve was prepared for the quantification of β-carotene (Additional file 1: Fig. S10). After the centrifugation at 8000*g* for 3 min, the β-carotene in dodecane layer was obtained and measured at 455 nm using UV spectrophotometer [8].

### Yeast intracellular ATP and ROS determination

The intracellular ATP was extracted and measured from the harvested yeast cells by using the ENLITEN^®^ ATP Assay System Bioluminescence Detection Kit (Promega, USA) according to the manufactures’ instructions. Briefly, after cultivated for 36 h, the cell pellets were washed twice and resuspended in PBS buffer to OD_600_ = 0.1 for ATP assays. Final concentration of 5% trichloroacetic acid (TCA) was required to extract ATP. Diluted 1 mL extract solution to 50 mL with Tris–Acetate buffer at pH 7.8, then measured in microwell plate-reading luminometer. The intracellular ROS was measured according to a previous study [49].

### iTRAQ-based proteomic analysis

After 36 h incubation in shake flask, yeast cells were collected by centrifugation at 10,000 rpm, 4 °C for 5 min and washed with cold PBS twice. Supernatant was removed, and cell pellets were frozen in liquid nitrogen, then stored at − 80 °C. For complete details of protein extraction, digestion, labeling with iTRAQ reagents, LC–MS/MS analysis, and data analysis, please see the Additional file 1: Additional methods section—*iTRAQ-based proteomic analysis*. Proteins displaying a 1.2-fold or more change between β-carotene producing strain YBX-01 and its parental strain YBX-B (*P* < 0.05) were considered as differential expressed proteins [50, 51].

### Membrane fluidity analysis

Cell membrane fluidity was assessed by measuring the fluorescence anisotropy of 1,6-diphenyl-1,3,5-hexatriene (DPH) [52]. Yeast cells were harvested and labeled with a 0.1 M potassium phosphate buffer (pH 6.8) and subsequently incubated in the dark at 25 °C for 40 min and 150 rpm. After incubation, cells were washed with the same buffer for three times. The fluorescence intensity was measured using a spectrofluorometer. Fluorescence anisotropy (*r*) values were calculated as described previously [53]. Decreases in the degree of fluorescence anisotropy represent increases in the fluidity of the cell membrane.

### Quantitative real-time PCR (qRT-PCR) analysis

Total RNA was isolated from the harvested yeast cells by using the HiPure Yeast RNA Kit (Magen, Guangzhou, China) as recommended in the manufacture’s protocol. Residual genomic DNA contamination was removed by an RNase-free DNase I treatment after RNA purification. The treated total RNA was reversely transcribed using HiScript^®^ II Q RT SuperMix for qPCR (+ gDNA wiper) (Vazyme, Nanjing, China). Specific primers for the analysis of gene expression were designed and used in qRT-PCR (Additional file 1: Table S3). The housekeeping gene *ACT1* was used as the reference gene to normalize the different samples. The relative gene expression analysis was performed using the 2^−ΔΔ*CT*^ method [54].

## Supplementary information

**Additional file 1: Fig. S1.** Changes in intracellular β-carotene content (A) and specific β-carotene production rate (B) in YBX-01.** Fig. S2.** β-Carotene secretion in additional ABC transporter overexpression strains.** Fig. S3.** Promoter replacement plasmid construction schematic (A) and yeast transformation schematic (B).** Fig. S4.** Genome PCR confirmation of ABC transporter overexpression strains.** Fig. S5.** The relative expression level of over-expressed ABC transporter genes.** Fig. S6. **Cell growth, glucose concentration and intracellular β-carotene concentration of YBX-01with or without dodecane.** Fig. S7.** Changes of gene expression level of the five transporters (A) and exported β-carotene level (B) in YBX-01 throughout the cell growth period.** Fig. S8.** Intracellular ROS determination in YBX-B and YBX-01 at 36 h.** Fig. S9.** The cell growth of YBX-SNQ2 with different ATP supply strategies.** Fig. S10.** The spectral characteristics (A) and standard curve of β-carotene in dodecane (B).** Table S1.** Comparative proteome analysis between YBX-01 and YBX-B.** Table S2.** Plasmids used in this study.** Table S3.** Primers used in this study.

## Data Availability

All data generated or analyzed during this study are included in this manuscript and its additional file.
