# Supplementary material for: Engineering endogenous ABC transporter with improving ATP supply and membrane flexibility enhances the secretion of β-carotene in Saccharomyces cerevisiae
Source: Biotechnol Biofuels. 2020 Oct 10;13:168. doi: 10.1186/s13068-020-01809-6 (PMC7548044; doi:10.1186/s13068-020-01809-6)
Supplement: Supplementary file 1 — Additional file 1: Fig. S1. Changes in intracellular β-carotene content (A) and specific β-carotene production rate (B) in YBX-01. Fig. S2. β-Carotene secretion in additional ABC transporter overexpression strains. Fig. S3. Promoter replacement plasmid construction schematic (A) and yeast transformation schematic (B). Fig. S4. Genome PCR confirmation of ABC transporter overexpression strains. Fig. S5. The relative expression level of over-expressed ABC transporter genes. Fig. S6. Cell growth, glucose concentration and intracellular β-carotene concentration of YBX-01with or without dodecane. Fig. S7. Changes of gene expression level of the five transporters (A) and exported β-carotene level (B) in YBX-01 throughout the cell growth period. Fig. S8. Intracellular ROS determination in YBX-B and YBX-01 at 36 h. Fig. S9. The cell growth of YBX-SNQ2 with different ATP supply strategies. Fig. S10. The spectral characteristics (A) and standard curve of β-carotene in dodecane (B). Table S1. Comparative proteome analysis between YBX-01 and YBX-B. Table S2. Plasmids used in this study. Table S3. Primers used in this study. [file 13068_2020_1809_MOESM1_ESM.doc]

**Additional file 1**

**Engineering endogenous ABC transporter with improving ATP supply and membrane flexibility enhances the secretion of β-carotene in *Saccharomyces cerevisiae***

Xiao Bu^1,2^, Jing-Yuan Lin^1,2^, Jing Cheng^1,2^, Dong Yang^3^, Chang-Qing Duan^1,2^, Mattheos Koffas^4^ and Guo-Liang Yan^1,2^ *

1. Centre for Viticulture and Enology, College of Food Science and Nutritional Engineering, China Agricultural University, Beijing 100083, China
2. Key Laboratory of Viticulture and Enology, Ministry of Agriculture and Rural Affairs, Beijing 100083, China
3. Beijing Key Laboratory of Functional Food from Plant Resources, College of Food Science and Nutritional Engineering, China Agricultural University, Beijing 100083, China
4. Center for Biotechnology and Interdisciplinary Studies and Department of Chemical and Biological Engineering, Rensselaer Polytechnic Institute, Troy, New York 12180, United States

* Corresponding author: Guo-Liang Yan

Tel: +86-10-62737039; Fax: +86-10-62738658; E-mail: [glyan@cau.edu.cn](mailto:glyan@cau.edu.cn)

Mailing address: 17 East Tsinghua Rd, College of Food Science and Nutritional Engineering, China Agricultural University, Beijing, 100083, China

Outline

| **No.** | **Title** |
| --- | --- |
| Fig. S1 | Changes in intracellular β-carotene content (A) and specific β-carotene production rate (B) in YBX-01 |
| Fig. S2 | β-Carotene secretion in additional ABC transporter overexpression strains |
| Fig. S3 | Promoter replacement plasmid construction schematic (A) and yeast transformation schematic (B) |
| Fig. S4 | Genome PCR confirmation of ABC transporter overexpression strains |
| Fig. S5 | The relative expression level of over-expressed ABC transporter genes |
| Fig. S6 | Cell growth, glucose concentration and intracellular β-carotene concentration in YBX-01with or without dodecane |
| Fig. S7 | Changes of gene expression level of the five transporters (A) and exported β-carotene level (B) throughout the cell growth period |
| Fig. S8 | Intracellular ROS determination in YBX-B and YBX-01 at 36 h |
| Fig. S9 | The cell growth of YBX-SNQ2 with different ATP supply strategies |
| Fig. S10 | The spectral characteristics (A) and standard curve of β-carotene in dodecane (B) |
| Table S1 | Comparative proteome analysis between YBX-01 and YBX-B |
| Table S2 | Plasmids used in this study |
| Table S3 | Primers used in this study |
| Methods | Promoter replacement plasmids construction |
|  | iTRAQ-based proteomic analysis |


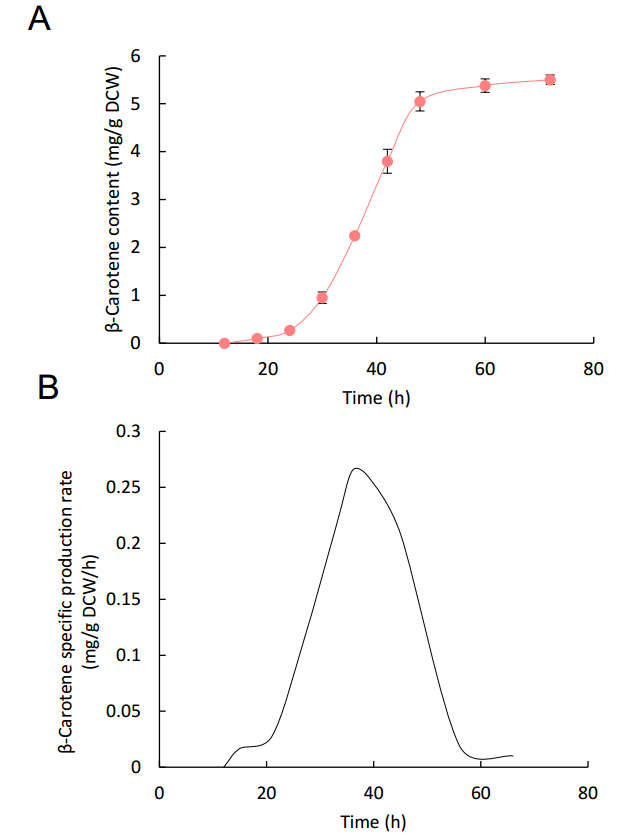


**Fig. S1.** Changes in intracellular β-carotene content (A) and specific β-carotene production rate (B) in YBX-01.


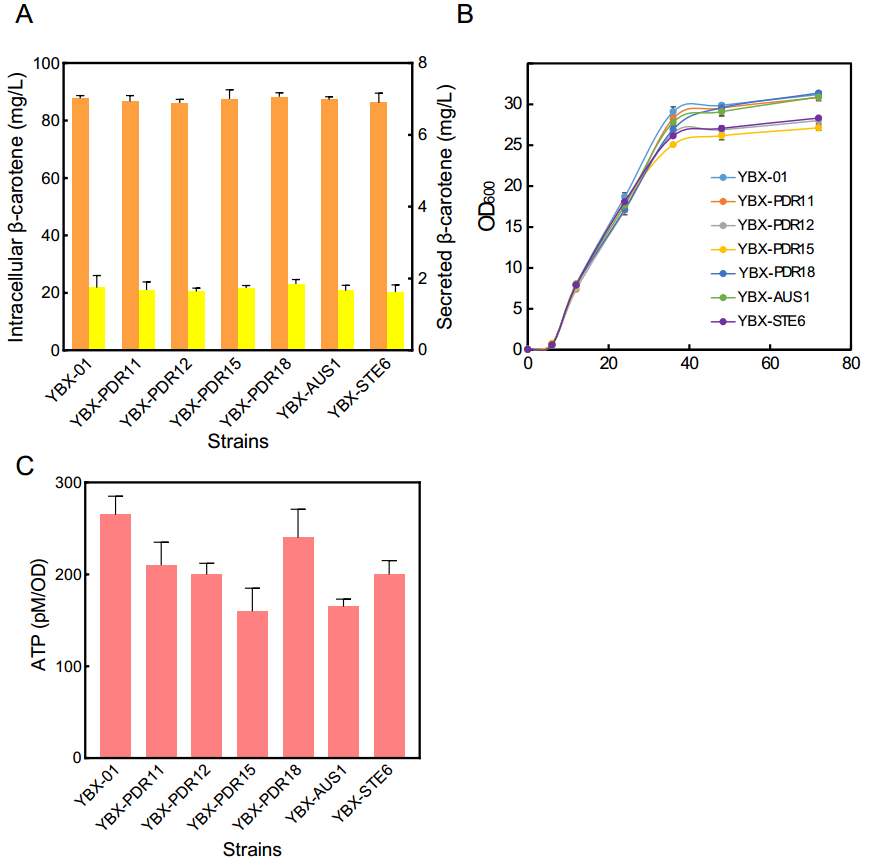


**Fig. S2.** β-Carotene secretion in additional ABC transporter overexpression strains. (A) The intracellular (in orange) and secreted (in yellow) β-carotene, (B) cell growth, and (C) intracellular ATP content of additional ABC transporter overexpression strains.


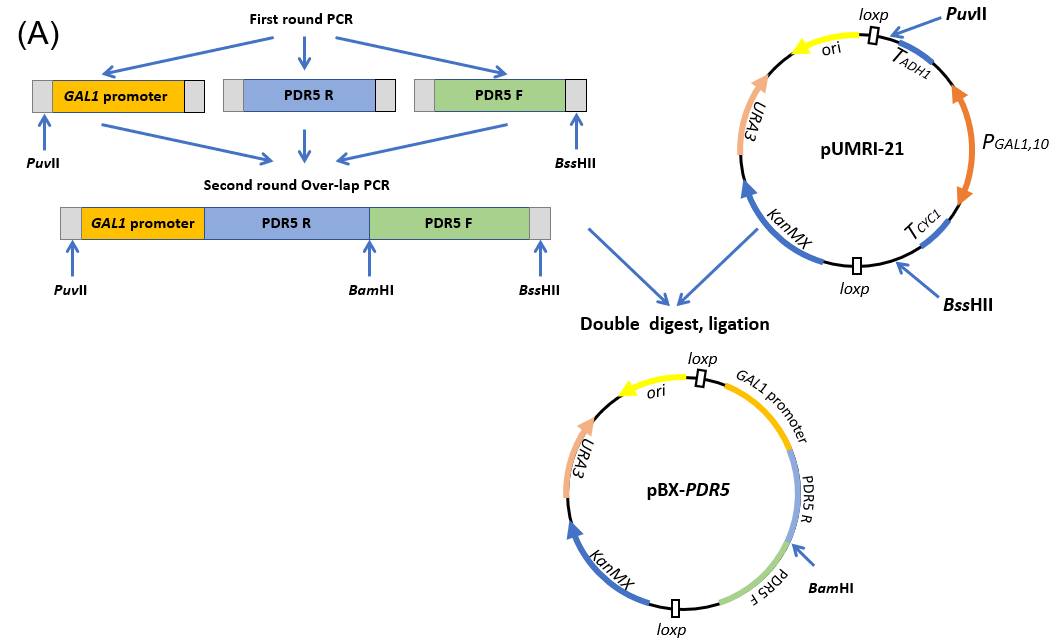

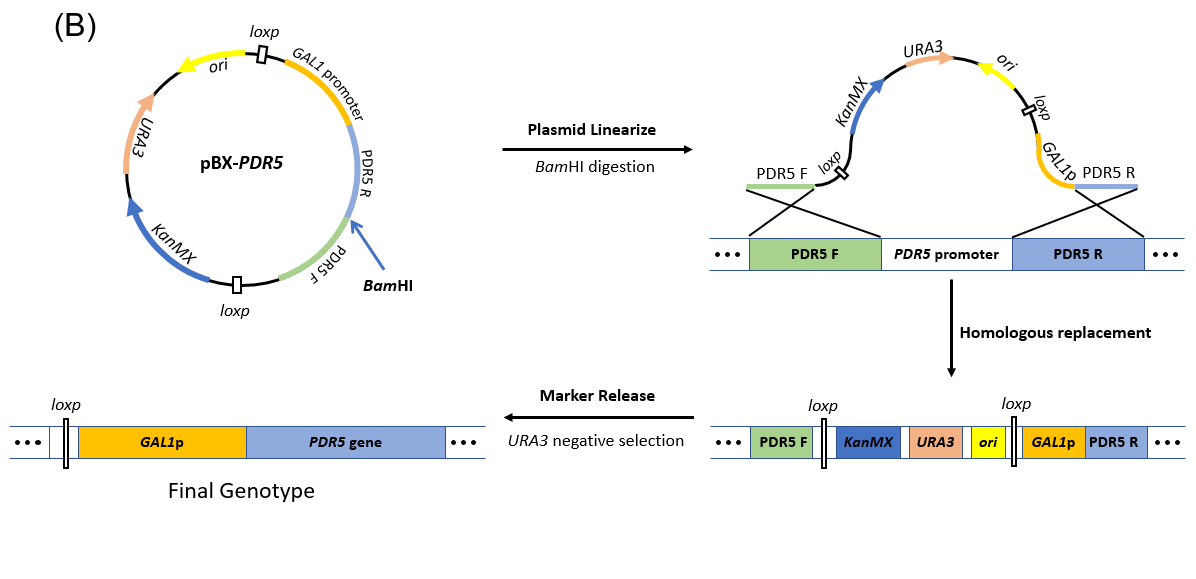


**Fig. S3.** Promoter replacement plasmid construction schematic (A) and yeast transformation schematic (B). Take pBX-*PDR5* as an example. The detailed construction procedure of the promoter replacement plasmids seen in **Additional Methods** (*Promoter replacement plasmids construction*). Before yeast transformation, the plasmid pBX-*PDR5* was linearized by *Bam*HI. Then, the linearized pBX-*PDR5* replaced the promoter of *PDR5* on the genome through the homologous arms (PDR5 F and PDR5 R). After the *loxp-KanMX-URA3-pbr322ori-loxp* region was removed by *URA3* negative selection, the promoter of *PDR5* was replaced by strong inducible promoter GAL1 successfully.


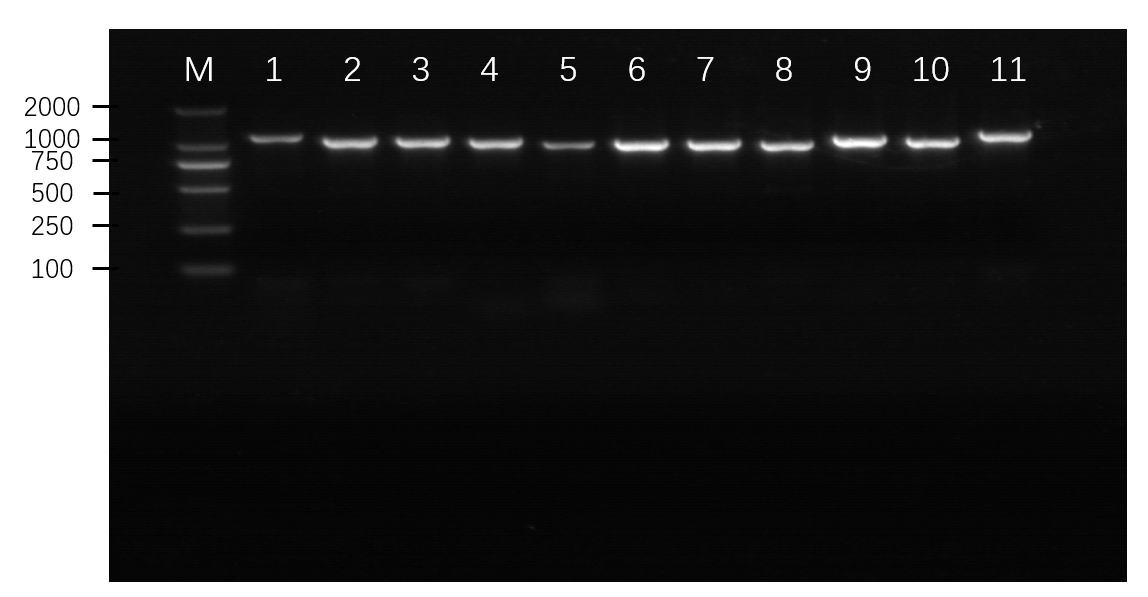


**Fig. S4.** Genome PCR confirmation of ABC transporter overexpression strains. M: Marker D2000; 1: YBX-PDR5; 2: YBX-PDR10; 3: YBX-PDR11; 4: YBX-PDR12; 5: YBX-PDR15; 6: YBX-PDR18; 7: YBX-SNQ2; 8: YBX-YOR1; 9: YBX-AUS1; 10: YBX-STE6; 11: YBX-YOL075C.


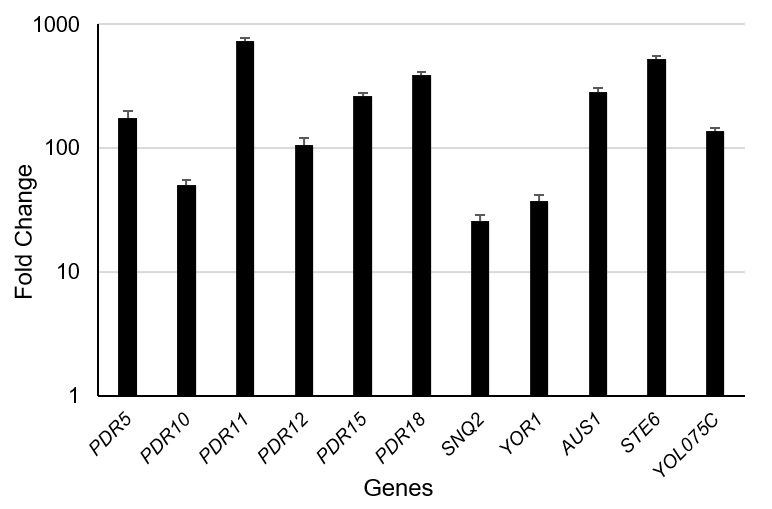


**Fig. S5.** The relative expression level of over-expressed ABC transporter genes. The qPCR analysis of gene expression levels of different yeast strains in which the native gene promoters were replaced by the strong inducible *GAL1* promoter at 36 h.


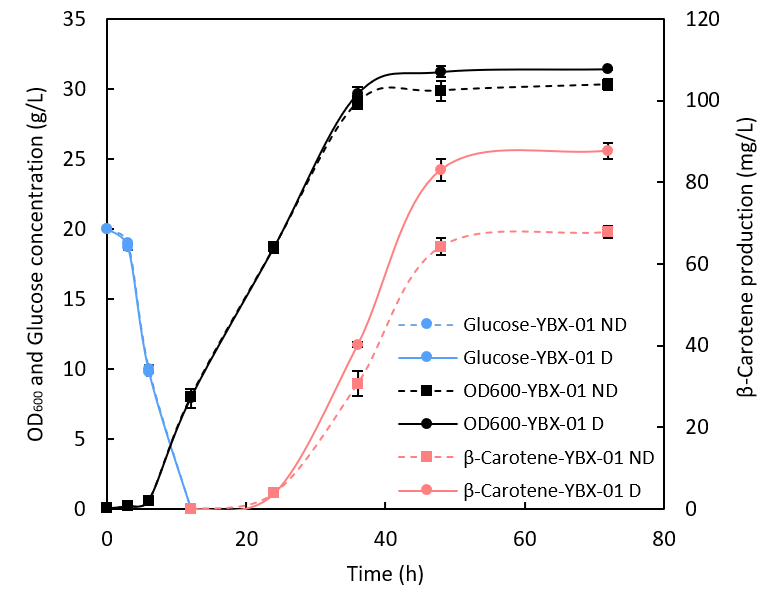


**Fig. S6.** Cell growth, glucose concentration and intracellular β-carotene content of YBX-01 with or without dodecane. ND: no dodecane; D: with dodecane.


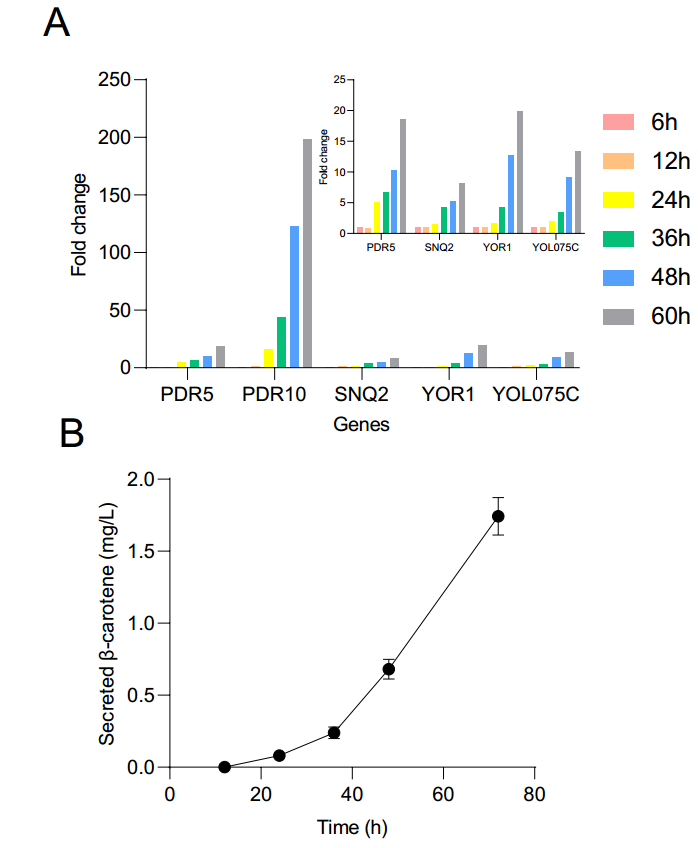


**Fig. S7.** Changes of gene expression level of the five transporters (A) and secreted β-carotene level (B) in YBX-01 throughout the cell growth period. The cells at 6 h were used as the control for qPCR analysis.


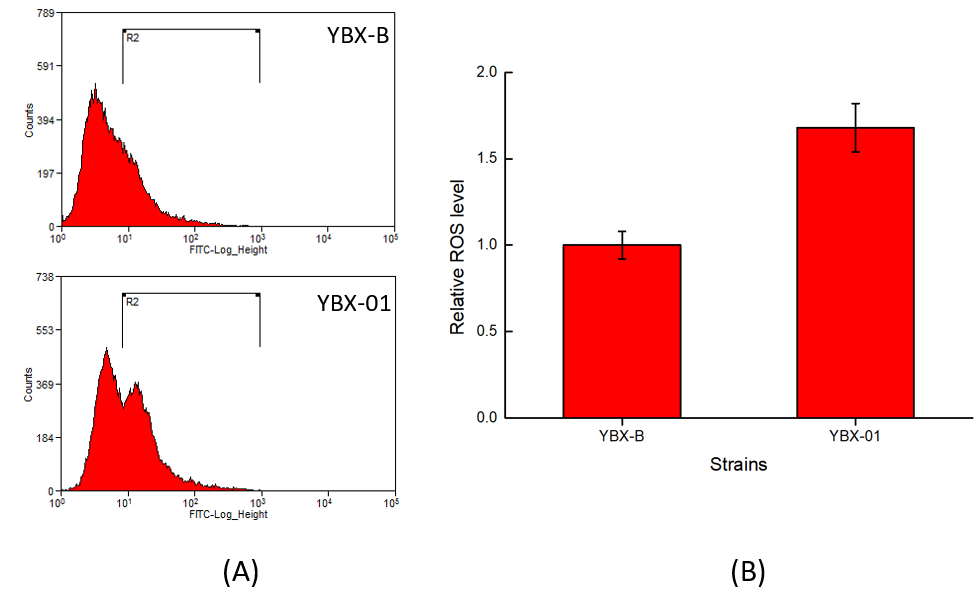


**Fig. S8.** Intracellular ROS determination in YBX-B and YBX-01 at 36 h.


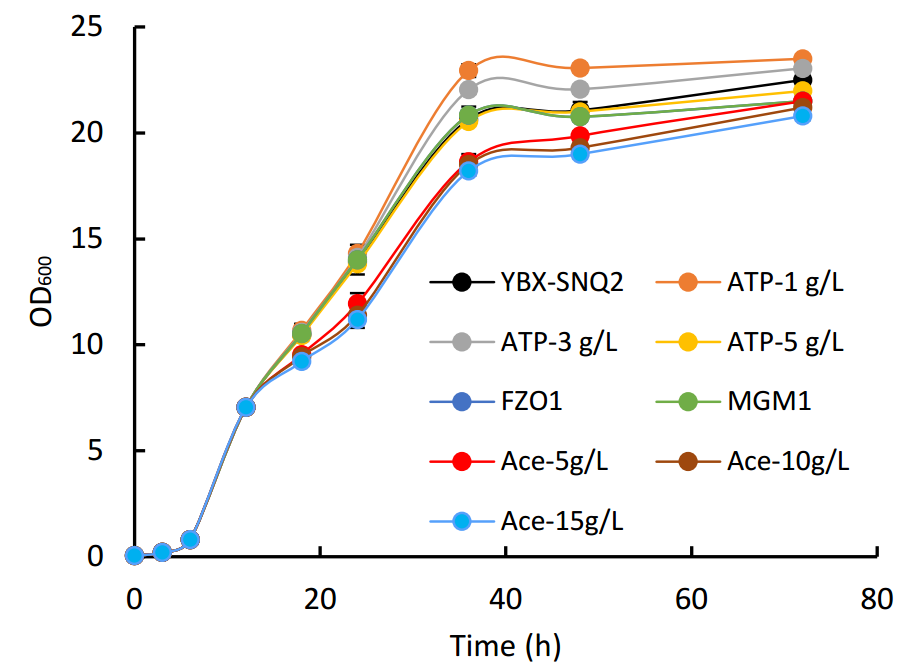


**Fig. S9.** The cell growth of YBX-SNQ2 with different ATP supply strategies.


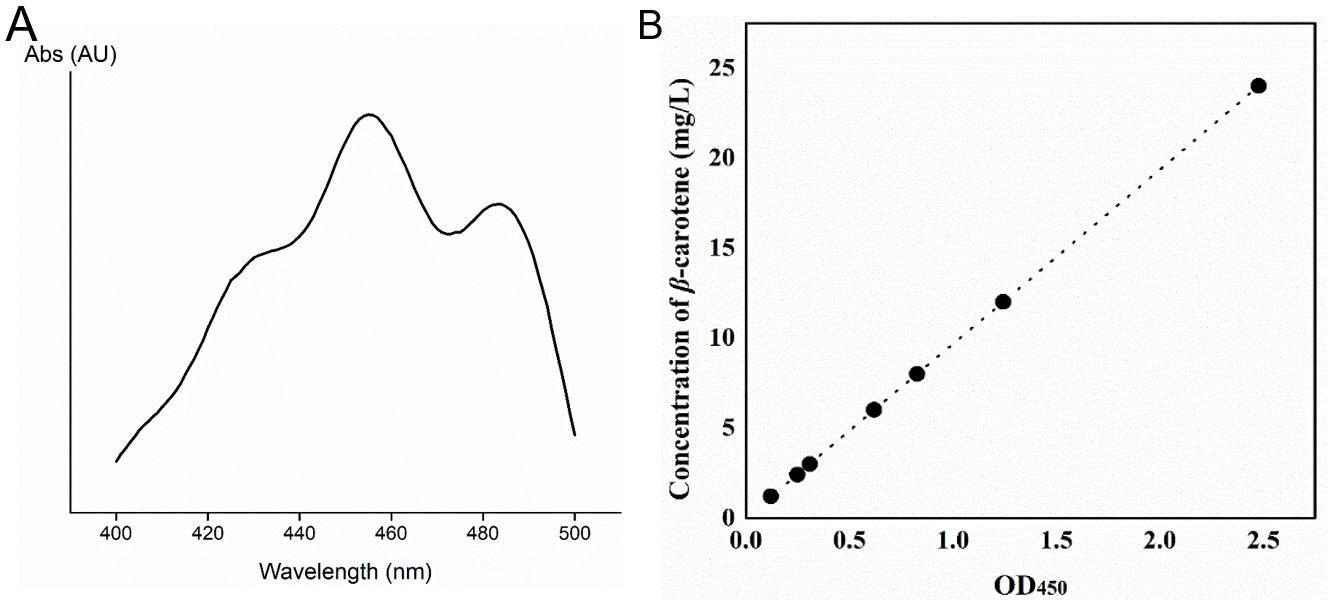


**Fig. S10.** The spectral characteristics (A) and standard curve of *β*-carotene in dodecane (B). Calculation equations: *β*-carotene concentration (mg/L) = 9.6713*OD450 + 0.0159, R^2^ = 0.9999.

**Table S1.** Comparative proteome analysis between YBX-01 and YBX-B.

| Category | Protein name | Protein ID | Fold change | P-value | UP/  DOWN |
| --- | --- | --- | --- | --- | --- |
| Carbohydrate metabolism | Pck1p | YKR097W | 1.368 | 0.010 | up |
|  | Dak1p | YML070W | 0.814 | 0.024 | down |
|  | Adh2p | YMR303C | 1.940 | 0.001 | up |
|  | Pgm2p | YMR105C | 1.525 | 0.019 | up |
|  | Glk1p | YCL040W | 1.286 | 0.002 | up |
|  | Adh6p | YMR318C | 1.656 | 0.001 | up |
|  | Gcy1p | YOR120W | 2.215 | 0.011 | up |
|  | Pdc5p | YLR134W | 0.613 | 0.002 | down |
|  | Gpp2p | YER062C | 0.743 | 0.001 | down |
|  | Gip2p | YER054C | 0.789 | 0.005 | down |
|  | Err3p | YMR323W | 0.737 | 0.003 | down |
|  | Nth2p | YBR001C | 1.246 | 0.040 | up |
| Amino acid metabolism | Ilv5p | YLR355C | 1.291 | 0.036 | up |
|  | Aro1p | YDR127W | 1.239 | 0.003 | up |
|  | Gdh3p | YAL062W | 0.798 | 0.011 | down |
|  | Gdh1p | YOR375C | 0.638 | 0.001 | down |
|  | Aro8p | YGL202W | 0.749 | 0.010 | down |
|  | Aro9p | YHR137W | 0.742 | 0.035 | down |
|  | Gln1p | YPR035W | 0.764 | 0.010 | down |
|  | Sam2p | YDR502C | 1.390 | 0.014 | up |
|  | Ser33p | YIL074C | 1.345 | 0.000 | up |
|  | Met17p | YLR303W | 1.578 | 0.008 | up |
|  | Arg1p | YOL058W | 1.230 | 0.002 | up |
|  | Bna5p | YLR231C | 1.298 | 0.007 | up |
|  | Aro4p | YBR249C | 1.256 | 0.020 | up |
|  | Hpa3p | YEL066W | 0.784 | 0.004 | down |
|  | Trp1p | YDR007W | 0.805 | 0.015 | down |
|  | Cha1p | YCL064C | 0.791 | 0.001 | down |
|  | Met1p | YKR069W | 1.417 | 0.010 | up |
| Cell wall synthesis | Mpg1p | YDL055C | 1.204 | 0.016 | up |
|  | Crh1p | YGR189C | 1.365 | 0.021 | up |
|  | Pst1p | YDR055W | 1.593 | 0.001 | up |
| Ergosterol biosynthetic process | Erg20p | YJL167W | 1.222 | 0.039 | up |
|  | Erg6p | YML008C | 1.288 | 0.033 | up |
|  | Erg1p | YGR175C | 2.112 | 0.001 | up |
|  | Erg25p | YGR060W | 1.647 | 0.043 | up |
|  | Erg24p | YNL280C | 1.527 | 0.007 | up |
|  | Erg4p | YGL012W | 1.329 | 0.005 | up |
| Energy metabolism | Ald4p | YOR374W | 1.307 | 0.001 | up |
|  | Sdh1p | YKL148C | 1.204 | 0.005 | up |
|  | Sdh2p | YLL041C | 1.298 | 0.027 | up |
|  | Lsc2p | YGR244C | 1.261 | 0.001 | up |
|  | Mdh3p | YDL078C | 1.205 | 0.003 | up |
|  | Tps3p | YMR261C | 0.806 | 0.012 | down |
|  | Lsc1p | YOR142W | 1.279 | 0.000 | up |
|  | Atp7p | YKL016C | 1.423 | 0.001 | up |
|  | Nde2p | YDL085W | 1.532 | 0.009 | up |
|  | Cit3p | YPR001W | 1.386 | 0.031 | up |
|  | Cit2p | YCR005C | 1.310 | 0.007 | up |
|  | Atp20p | YPR020W | 1.325 | 0.020 | up |
|  | Tim11p | YDR322C-A | 1.467 | 0.003 | up |
|  | Coa1p | YIL157C | 1.363 | 0.006 | up |
|  | Ppa2p | YMR267W | 1.234 | 0.027 | up |
|  | Atp17p | YDR377W | 1.432 | 0.005 | up |
|  | Sdh3p | YKL141W | 1.223 | 0.014 | up |
|  | Som1p | YEL059C-A | 1.517 | 0.005 | up |
|  | Atp19p | YOL077W-A | 1.349 | 0.010 | up |
| Lipid metabolism | Acc1p | YNR016C | 1.332 | 0.010 | up |
|  | Fox2p | YKR009C | 1.316 | 0.012 | up |
|  | Faa1p | YOR317W | 0.832 | 0.004 | down |
|  | Pox1p | YGL205W | 1.536 | 0.026 | up |
|  | Ino1p | YJL153C | 2.118 | 0.005 | up |
|  | Sps19p | YNL202W | 1.279 | 0.005 | up |
|  | Faa2p | YER015W | 1.299 | 0.034 | up |
|  | Oar1p | YKL055C | 1.215 | 0.047 | up |
|  | Eci1p | YLR284C | 1.515 | 0.001 | up |
|  | Cyb5p | YNL111C | 1.391 | 0.022 | up |
| Mitochondrial translation | Mrps35p | YGR165W | 1.282 | 0.010 | up |
|  | Mrp51p | YPL118W | 1.483 | 0.009 | up |
|  | Mrps18p | YNL306W | 1.252 | 0.002 | up |
|  | Rsm28p | YDR494W | 1.314 | 0.043 | up |
|  | Mrpl8p | YJL063C | 1.258 | 0.008 | up |
|  | Rsm23p | YGL129C | 1.387 | 0.016 | up |
|  | Mrpl28p | YDR462W | 1.310 | 0.010 | up |
|  | Nam9p | YNL137C | 1.307 | 0.030 | up |
|  | Tma10p | YLR327C | 1.205 | 0.048 | up |
|  | Rsm7p | YJR113C | 1.329 | 0.005 | up |
|  | Mrpl27p | YBR282W | 1.565 | 0.001 | up |
|  | Pet112p | YBL080C | 1.258 | 0.046 | up |
|  | Atp13p | YMR282C | 1.248 | 0.021 | up |
|  | Rn49p | YJL096W | 1.364 | 0.030 | up |
|  | Mrpl51p | YPR100W | 1.305 | 0.038 | up |
|  | Mrp21p | YBL090W | 1.393 | 0.000 | up |
|  | Pet123p | YOR158W | 1.543 | 0.002 | up |
|  | Rpm2p | YML091C | 1.561 | 0.010 | up |
|  | Mrp17p | YKL003C | 1.362 | 0.018 | up |
| Transport | Pma2p | YPL036W | 1.461 | 0.000 | up |
|  | Pdr5p | YOR153W | 1.201 | 0.010 | up |
|  | Hxt6p | YDR343C | 0.475 | 0.021 | down |
|  | Pdr12p | YPL058C | 0.766 | 0.003 | down |
|  | Gal2p | YLR081W | 1.795 | 0.003 | up |
|  | Odc1p | YPL134C | 1.343 | 0.010 | up |
|  | Ady2p | YCR010C | 1.443 | 0.016 | up |
|  | Pdr10p | YOR328W | 1.257 | 0.017 | up |
|  | Yhm2p | YMR241W | 1.302 | 0.030 | up |
|  | Erp3p | YDL018C | 1.211 | 0.009 | up |
|  | Cot1p | YOR316C | 0.753 | 0.002 | down |
|  | Mup1p | YGR055W | 1.896 | 0.040 | up |
|  | Smf1p | YOL122C | 1.290 | 0.023 | up |
|  | Chs3p | YBR023C | 0.617 | 0.003 | down |
|  | Chs6p | YJL099W | 1.242 | 0.016 | up |
|  | Alr1p | YOL130W | 1.319 | 0.044 | up |
|  | Pxa1p | YPL147W | 1.333 | 0.014 | up |
|  | Fre1p | YLR214W | 1.319 | 0.001 | up |
|  | Rsn1p | YMR266W | 0.798 | 0.041 | down |

**Table S2.** Plasmids used in this study.

| Plasmid | Description | Source |
| --- | --- | --- |
| YIplac211YB/I/E* | YIplac211 *P_TDH3_-crtYB-T_CYC1_*; *P_TDH3_-crtI-T_CYC1_*; *P_TDH3_-crtE-T_CYC1_* | Verwaal et al., 2007 |
| pUMRI-21 | No Homologus Arm (HA)  *loxp-KanMX-URA3-pbr322ori-loxp, T_ADH1_-MCS1-P_GAL10_-P_GAL1_-MCS2-T_CYC1_* | Lv et al., 2016 (KM216411) |
| pBX*-CrtI-CrtYB* | *CrtI* cloned into MCS1 and *CrtYB* cloned into MCS2 of pUMRI-21 | This study |
| pBX-*CrtE* | *CrtE* cloned into MCS1 of pUMRI-21 | This study |
| pBX*-CrtI-CrtYB*  -*gal1-10-7* | Gal1-10-7 HA, *T_ADH1_*- *CrtI* -*P_GAL10_*-*P_GAL1_*- *CrtYB* - *T_CYC1_* | This study |
| pBX-*CrtE*-*gal80* | Gal80 HA, *T_ADH1_*-*CrtE*-*P_GAL10_*-*P_GAL1_*-*MCS2*-*T_CYC1_* | This study |
| pBX-*PDR5* | The *GAL1* promoter (*PDR5*), PDR5 R, and PDR5 F were ligated together by overlap method, then infused with *Puv*II/*Bss*HII digested pUMRI-21 plasmid which contains the *loxp-kanMX-URA3-pbr322ori-loxp* region | This study  (Fig. S1A) |
| pBX-*PDR10* | The *GAL1* promoter (*PDR10*), PDR10 R, and PDR10 F were ligated together by overlap method, then infused with *Puv*II/*Bss*HII digested pUMRI-21 plasmid which contains the *loxp-kanMX-URA3-pbr322ori-loxp* region | This study |
| pBX-*PDR11* | The *GAL1* promoter (*PDR11*), PDR11 R, and PDR11 F were ligated together by overlap method, then infused with *Puv*II/*Bss*HII digested pUMRI-21 plasmid which contains the *loxp-kanMX-URA3-pbr322ori-loxp* region | This study |
| pBX-*PDR12* | The *GAL1* promoter (*PDR12*), PDR12 R, and PDR12 F were ligated together by overlap method, then infused with *Puv*II/*Bss*HII digested pUMRI-21 plasmid which contains the *loxp-kanMX-URA3-pbr322ori-loxp* region | This study |
| pBX-*PDR15* | The *GAL1* promoter (*PDR15*), PDR15 R, and PDR15 F were ligated together by overlap method, then infused with *Puv*II/*Bss*HII digested pUMRI-21 plasmid which contains the *loxp-kanMX-URA3-pbr322ori-loxp* region | This study |
| pBX-*PDR18* | The *GAL1* promoter (*PDR18*), PDR18 R, and PDR18 F were ligated together by overlap method, then infused with *Puv*II/*Bss*HII digested pUMRI-21 plasmid which contains the *loxp-kanMX-URA3-pbr322ori-loxp* region | This study |
| pBX-*SNQ2* | The *GAL1* promoter (*SNQ2*), SNQ2 R, and SNQ2 F were ligated together by overlap method, then infused with *Puv*II/*Bss*HII digested pUMRI-21 plasmid which contains the *loxp-kanMX-URA3-pbr322ori-loxp* region | This study |
| pBX-*YOR1* | The *GAL1* promoter (*YOR1*), YOR1 R, and YOR1 F were ligated together by overlap method, then infused with *Puv*II/*Bss*HII digested pUMRI-21 plasmid which contains the *loxp-kanMX-URA3-pbr322ori-loxp* region | This study |
| pBX-*AUS1* | The *GAL1* promoter (*AUS1*), AUS1 R, and AUS1 F were ligated together by overlap method, then infused with *Puv*II/*Bss*HII digested pUMRI-21 plasmid which contains the *loxp-kanMX-URA3-pbr322ori-loxp* region | This study |
| pBX-*STE6* | The *GAL1* promoter (*STE6*), STE6 R, and STE6 F were ligated together by overlap method, then infused with *Puv*II/*Bss*HII digested pUMRI-21 plasmid which contains the *loxp-kanMX-URA3-pbr322ori-loxp* region | This study |
| pBX-*YOL075C* | The *GAL1* promoter (*YOL075C*), YOL075C R, and YOL075C F were ligated together by overlap method, then infused with *Puv*II/*Bss*HII digested pUMRI-21 plasmid which contains the *loxp-kanMX-URA3-pbr322ori-loxp* region | This study |
| pBX-*FZO1* | The *GAL1* promoter (*FZO1*), FZO1 R, and FZO1 F were ligated together by overlap method, then infused with *Puv*II/*Bss*HII digested pUMRI-21 plasmid which contains the *loxp-kanMX-URA3-pbr322ori-loxp* region | This study |
| pBX-*MGM1* | The *GAL1* promoter (*MGM1*), MGM1 R, and MGM1 F were ligated together by overlap method, then infused with *Puv*II/*Bss*HII digested pUMRI-21 plasmid which contains the *loxp-kanMX-URA3-pbr322ori-loxp* region | This study |
| pBX-*OLE1* | The *TEF1* promoter (*OLE1*), OLE1 R, and OLE1 F were ligated together by overlap method, then infused with *Puv*II/*Bss*HII digested pUMRI-21 plasmid which contains the *loxp-kanMX-URA3-pbr322ori-loxp* region | This study |

MCS: Multiple cloning site.

All plasmids except YIplac211YB/I/E* share the same structure of *loxp-kanMX-URA3-pbr322ori-loxp*.

**Table S3.** Primers used in this study.

| Primers | Sequences (5’-3’) | Note |
| --- | --- | --- |
| **For amplification of structural genes** | | |
| CrtYB F (KpnI) | AGAAGACCTCGAGTAAGCTTGGTACATGACGGCTCTCGCATATTAC | *CrtYB* ORF |
| CrtYB R (KpnI) | AGCGGATCTTAGCTAGCCGCGGTACACTTACTGCCCTTCCCATCC |
| CrtI F (EcoRI) | GTAAGAATTTTTGAAAATTCGAATTATGGGAAAAGAACAAGATCAGGATAA | *CrtI* ORF |
| CrtI R (EcoRI) | GCCGCCCTTTAGTGAGGGTTGAATTTCAGAAAGCAAGAACACCAACG |
| CrtE F (EcoRI) | GTAAGAATTTTTGAAAATTCGAATTATGGATTACGCGAACATCCT | *CrtE* ORF |
| CrtE R (EcoRI) | GCCGCCCTTTAGTGAGGGTTGAATTTCACAGAGGGATATCGGCTAG |
| **For amplification of homologous arms** | | |
| GAL1-10-7 left F | GAAGCTTGCCTCAATTAGCCGGCCATATAGGCCGTCAATCTCTGGACAAGAACATTC | *GAL1-10-7* homologous arm |
| GAL1-10-7 left R | AGGGAACAAAAGCTGGAGCTGGCCGAGTGGTAGAAGTCACTACAGC |
| GAL1-10-7 right F | GGCGTAATAGCGAAGAGGCCGAGCTACTGGTAACCTAAACCC |
| GAL1-10-7 right R | GAATGTTCTTGTCCAGAGATTGACGGCCTATATGGCCGGCTAATTGAGGCAAGCTTC |
| GAL80 left F | AAAAGTGAGAGAAGGTGCACGGCCATATAGGCCCATATCACTGCTGGTCCTTG | *GAL80* homologous arm |
| GAL80 left R | AGGGAACAAAAGCTGGAGCTGGCCAGATGATGGTTTGAACCCCA |
| GAL80 right F | GGCGTAATAGCGAAGAGGCCGTGGAACTAGAGCAAACGAC |
| GAL80 right R | CAAGGACCAGCAGTGATATGGGCCTATATGGCCGTGCACCTTCTCTCACTTTT |
| **For promoter replacement plasmids construction** | | |
| GAL1 F* | CCTCTTCGCTATTACGCCAGTGAAGTACGGATTAGAAGCCG |  |
| GAL1 R (PDR5) | TTATTGTTAAGCTTGGCCTCGGGCATCTCCTTGACGTTAAAGTATAGAGG | P*_GAL1_* (*PDR5*) promoter |
| GAL1 R (PDR10) | CTTGAGGGCGCTTGCAACATTCTCCTTGACGTTAAAGTATAGAGG | P*_GAL1_* (*PDR10*) promoter |
| GAL1 R (PDR11) | ATATTTGGAAAGAGACATCCGCCTCCTTGACGTTAAAGTATAGAGG | P*_GAL1_* (*PDR11*) promoter |
| GAL1 R (PDR12) | TGTTCGTCAGTCGAAGACATCTCCTTGACGTTAAAGTATAGAGG | P*_GAL1_* (*PDR12*) promoter |
| GAL1 R (PDR15) | TCTACGTCTCTGATATCTGATGACATTTTTCTCCTTGACGTTAAAGTATAGAG | P*_GAL1_* (*PDR15*) promoter |
| GAL1 R (PDR18) | CTTCTACTGAAACGCATTCCATTCTCCTTGACGTTAAAGTATAGAGG | P*_GAL1_* (*PDR18*) promoter |
| GAL1 R (SNQ2) | CGTGCTTTTGATATTGCTCATTTTTCTCCTTGACGTTAAAGTATAGAG | P*_GAL1_* (*SNQ2*) promoter |
| GAL1 R (YOR1) | TCCCCCACGGTAATCGTCATCTCCTTGACGTTAAAGTATAGAGG | P*_GAL1_* (*YOR1*) promoter |
| GAL1 R (AUS1) | GAGTGAAGTACTTTGAAATTGACATCTCCTTGACGTTAAAGTATAGAGG | P*_GAL1_* (*AUS1*) promoter |
| GAL1 R (STE6) | TGTTTTGTAGTCTTAAAACTTAAAAAGTTCATTCTCCTTGACGTTAAAGTATAGAGG | P*_GAL1_* (*STE6*) promoter |
| GAL1 R (YOL075C) | CCATTCTCCTGCTGTGACATATCTCCTTGACGTTAAAGTATAGAGG | P*_GAL1_* (*YOL075C*) promoter |
| GAL1 R (FZO1) | AATTGTTGTTTTCCTTCAGACATTCTCCTTGACGTTAAAGTATAGAGG | P*_GAL1_* (*FZO1*) promoter |
| GAL1 R (MGM1) | GGCTCGCATTCATCCTTACTAACTCCTTGACGTTAAAGTATAGAGG | P*_GAL1_* (*MGM1*) promoter |
| TEF1 F (OLE1) | CCTCTTCGCTATTACGCCAGCACACACCATAGCTTCAAAATG | P*_TEF1_* (*OLE1*) promoter |
| TEF1 R (OLE1) | AGTAGTTCCAGAAGTTGGCATTTGTAATTAAAACTTAGATTAGATTGCTATGC |
| PDR5 FF | GGTAGACCAGGCTCTGGCTGTACTGGATCCTTGGCGCAGTCCCTTACATAGT | PDR5 F |
| PDR5 FR | GTTATATTAAGGGTTGTCGAGCGCGCAGAGTTCCGCGGAGACATTTC |
| PDR5 RF | CCTCTATACTTTAACGTCAAGGAGATGCCCGAGGCCAAGCTTAACAATAA | PDR5 R |
| PDR5 RR | ACTATGTAAGGGACTGCGCCAAGGATCCAGTACAGCCAGAGCCTGGTCTACC |
| PDR10 FF | GAGTTGGAGGTGCATATCCCGACGAATTCCGCCTGAGTTACTCTCCTCTGG | PDR10 F |
| PDR10 FR | GTTATATTAAGGGTTGTCGAGCGCGCCGGGCGGCTGTAACAATGAC |
| PDR10 RF | CCTCTATACTTTAACGTCAAGGAGAATGTTGCAAGCGCCCTCAAG | PDR10 R |
| PDR10 RR | CCAGAGGAGAGTAACTCAGGCGGAATTCGTCGGGATATGCACCTCCAACTC |
| PDR11 FF | GGTGAAAGGAAACGTATCTCCGGATCCTCCACTTTGACGCCCCTTTA | PDR11 F |
| PDR11 FR | ATATTAAGGGTTGTCGAGCGCGCAGAGAGCTTTACGATGTTG |
| PDR11 RF | CCTCTATACTTTAACGTCAAGGAGGCGGATGTCTCTTTCCAAATAT | PDR11 R |
| PDR11 RR | TAAAGGGGCGTCAAAGTGGAGGATCCGGAGATACGTTTCCTTTCACC |
| PDR12 FF | CAAAATTGTACGGGTGTCGTGGATCCAGTGGCCTCTAAACCAAAGAT | PDR12 F |
| PDR12 FR | ATATTAAGGGTTGTCGAGCGCGCATAAGAACCGTCGAGAAAATGT |
| PDR12 RF | CCTCTATACTTTAACGTCAAGGAGATGTCTTCGACTGACGAACA | PDR12 R |
| PDR12 RR | ATCTTTGGTTTAGAGGCCACTGGATCCACGACACCCGTACAATTTTG |
| PDR15 FF | GCCGATGTGTCATACCAGTCGGATCCTGGCTGTAGAACATGCCTTAC | PDR15 F |
| PDR15 FR | ATATTAAGGGTTGTCGAGCGCGCTACAACAAGACTAGCAAATCTCT |
| PDR15 RF | CCTCTATACTTTAACGTCAAGGAGAAAAATGTCATCAGATATCAGAGACGTAGA | PDR15 R |
| PDR15 RR | GTAAGGCATGTTCTACAGCCAGGATCCGACTGGTATGACACATCGGC |
| PDR18 FF | ACTGCCAATAGAGAATTCTATGCGGATCCGACTCACCTGGTAAAACATCTATG | PDR18 F |
| PDR18 FR | ATATTAAGGGTTGTCGAGCGCGGTGTAAAATATCAGAATTCCCGG |
| PDR18 RF | CCTCTATACTTTAACGTCAAGGAGAATGGAATGCGTTTCAGTAGAAG | PDR18 R |
| PDR18 RR | CATAGATGTTTTACCAGGTGAGTCGGATCCGCATAGAATTCTCTATTGGCAGT |
| SNQ2 FF | GAGTGCCCTAGAAGGTGCTAGGATCCGCCAGACTATGTATGACTCG | SNQ2 F |
| SNQ2 FR | GTTATATTAAGGGTTGTCGAGCGCGCTCTCAGGGAGCTTGAACTCTA |
| SNQ2 RF | CTCTATACTTTAACGTCAAGGAGAAAAATGAGCAATATCAAAAGCACG | SNQ2 R |
| SNQ2 RR | CGAGTCATACATAGTCTGGCGGATCCTAGCACCTTCTAGGGCACTC |
| YOR1 FF | GAACGATACAGCCGAACGATCTCGGATCCGGAAGAAGGAAGTTTAGTGCCACC | YOR1 F |
| YOR1 FR | ATATTAAGGGTTGTCGAGCGCGGCGGAGAGCTGTTCTACCTCC |
| YOR1 RF | CCTCTATACTTTAACGTCAAGGAGATGACGATTACCGTGGGGGA | YOR1 R |
| YOR1 RR | GGTGGCACTAAACTTCCTTCTTCCGGATCCGAGATCGTTCGGCTGTATCGTTC |
| AUS1 FF | AATTCGGCCTGTCTCATGTAGGATCCAAAGTAAGCCATACGAACGG | AUS1 F |
| AUS1 FR | ATATTAAGGGTTGTCGAGCGCGAGAACTGTTCAGTGCCTTAGA |
| AUS1 RF | CCTCTATACTTTAACGTCAAGGAGATGTCAATTTCAAAGTACTTCACTC | AUS1 R |
| AUS1 RR | CCGTTCGTATGGCTTACTTTGGATCCTACATGAGACAGGCCGAATT |
| STE6 FF | ATTTAGTTGCAATATGTGCGCGGATCCCTATGACTTCCATAGCTCACC | STE6 F |
| STE6 FR | ATATTAAGGGTTGTCGAGCGCGTGCCCTCTGTGGGAATTGAAC |
| STE6 RF | CCTCTATACTTTAACGTCAAGGAGAATGAACTTTTTAAGTTTTAAGACTACAAAACA | STE6 R |
| STE6 RR | GGTGAGCTATGGAAGTCATAGGGATCCGCGCACATATTGCAACTAAAT |
| YOL075C FF | CTCTTCTGAGCGAACCAAGAAGGATCCACCATTGGAGAATACCGTTTG | YOL075C F |
| YOL075C FR | ATATTAAGGGTTGTCGAGCGCGCTTGTGCCTACTTCTGCATT |
| YOL075C RF | CCTCTATACTTTAACGTCAAGGAGATATGTCACAGCAGGAGAATGG | YOL075C R |
| YOL075C RR | CAAACGGTATTCTCCAATGGTGGATCCTTCTTGGTTCGCTCAGAAGAG |
| FZO1 FF | AAGCTAGATGGTCAGTACAATACGGATCCACTACCATCCTTCTAGCCTT | FZO1 F |
| FZO1 FR | GTTATATTAAGGGTTGTCGAGCGCGCGCTACTGTAACCTGCGTTATC |
| FZO1 RF | CCTCTATACTTTAACGTCAAGGAGAATGTCTGAAGGAAAACAACAATT | FZO1 R |
| FZO1 RR | AAGGCTAGAAGGATGGTAGTGGATCCGTATTGTACTGACCATCTAGCTT |
| MGM1 FF | ATAAGTCACAAGACGGTGGTGGATCCCGACAAGTAAGCTGTTCTTCT | MGM1 F |
| MGM1 FR | GTTATATTAAGGGTTGTCGAGCGCGCACTGGTGTGAACCTTCCTAG |
| MGM1 RF | CCTCTATACTTTAACGTCAAGGAGTTAGTAAGGATGAATGCGAGCC | MGM1 R |
| MGM1 RR | AGAAGAACAGCTTACTTGTCGGGATCCACCACCGTCTTGTGACTTAT |
| OLE1 FF | GCTCTCTCTGGTAAAGTGCCGGATCCTGCACGTCAAGATTCTCCGT | OLE1 F |
| OLE1 FR | ATATTAAGGGTTGTCGAGCGCGTCCACCTTTGTGTGCCATCC |
| OLE1 RF | GCATAGCAATCTAATCTAAGTTTTAATTACAAATGCCAACTTCTGGAACTACT | OLE1 R |
| OLE1 RR | ACGGAGAATCTTGACGTGCAGGATCCGGCACTTTACCAGAGAGAGC |
| **For quantitative RT-PCR** | | |
| qACT1-F | TGCAAAAGGAAATCACCGC | 98 bp |
| qACT1-R | GATAGAACCACCAATCCAGACG |
| qCrtYB-F | TGTTCTGGGTCTGTCTGCCTG | 109 bp |
| qCrtYB-R | CGGTGTAATGAGGGGAAATGA |
| qCrtI-F | CTGGCTTCGCAGCATTCTTA | 118 bp |
| qCrtI-R | ACTCTTCGTAATCTGTCGGTCTTG |
| qCrtE-F | GGATTCCGCAGACAATAAACAC | 120 bp |
| qCrtE-R | GAAGCGAAGCAGATGAAGGAG |
| qPDR5-F | GTGGTGTTATGACTACCCCAAGT | 185 bp |
| qPDR5-R | CATGTACTGCCCACATGTCATA |
| qPDR10-F | TGCTCTGCTATCGGTAGGACT | 150 bp |
| qPDR10-R | CTCCCATCGAGCAGATAACC |
| qPDR11-F | TGGGAAACCCAACATCAGCG | 190 bp |
| qPDR11-R | ATGGTTTGCTCCACCGTCAG |
| qPDR12-F | AGCCCTTGCCTGCTTCTTTC | 104 bp |
| qPDR12-R | CTCGGCTGCAAATCTCAGGG |
| qPDR15-F | GGCGTGGGTACAGAACATGG | 124 bp |
| qPDR15-R | CTGGTATGACACATCGGCGG |
| qPDR18-F | TTGGCTCAAAGGAACGTGGG | 178 bp |
| qPDR18-R | GAATAGACTGTGGCCGACGC |
| qSNQ2-F | CACAACCTGTCTCATTGATGC | 181 bp |
| qSNQ2-R | GGTTTCATGTACTCTCCACACG |
| qYOR1-F | GCCCGAAATGACACCTCCAG | 160 bp |
| qYOR1-R | AGCACCTGTACGACCACAGA |
| qAUS1-F | CGGCAGGGAAAGATACAGCG | 149 bp |
| qAUS1-R | ATCAACACCAGACGCACGAG |
| qSTE6-F | TCCTGACGGGCAGAGTGTTC | 130 bp |
| qSTE6-R | ATTACTGGCACAGAAGCCGC |
| qYOL075C-F | GGCAGTTATGGGTGGTTCGG | 181 bp |
| qYOL075C-R | CTGGATGGGATGGTCTTGGC |
| qFZO1-F | AGCAGCGTTTGCTACCTGAG | 133 bp |
| qFZO1-R | GCTTCCTTGAGAGTCGGAGC |
| qMGM1-F | CGTTCCGACTTCAGAGGCTG | 119 bp |
| qMGM1-R | GGTCTGCCGCTTCCACTTG |
| qOLE1-F | TCTACTACGCTGTCGGTGGT | 131 bp |
| qOLE1-R | GGACCCTTCAACGGAAGCAC |
| **For genomic PCR confirmation** | | |
| YZJYZ GAL1 F | TTCCTGAAACGCAGATGTGC | |
| YZJYZ PDR5 R | GATGTACATCAGCTTCTGCG | |
| YZJYZ PDR10 R | TCAGCAGCGTAGTACATCCT | |
| YZJYZ PDR11 R | ACCGATCTTGTAGCTTTTGC | |
| YZJYZ PDR12 R | TGGTCCAGACCATCATAGGA | |
| YZJYZ PDR15 R | CCATCCATGGGTTTCAGGAT | |
| YZJYZ PDR18 R | CCTTTCGCTGCTAATGCTTC | |
| YZJYZ SNQ2 R | CCAAAATCATTTCACCCGCT | |
| YZJYZ YOR1 R | CCTCTTCTTCTGTCGCTTCTG | |
| YZJYZ AUS1 R | GAGAGCTGTGGCGGAATCTA | |
| YZJYZ STE6 R | GGCTGCTTTACTCGTTTCAG | |
| YZJYZ YOL075C R | GTCTTCTCTTTTCACCACCAG | |
| YZJYZ FZO1 R | GTCACTGATACGATCTGAGAGT | |
| YZJYZ MGM1 R | CGTCATCGTCGAGTGATGTA | |
| YZJYZ TEF1 F | CGGTCTTCAATTTCTCAAGTTT | |
| YZJYZ OLE1 R | GGTATCAGTGTAACGATGGTG | |

* GAL1F is a common primer required for amplification of several promoters from yeast genomic DNA, including promoter P*_GAL1_* (*PDR5*), P*_GAL1_* (*PDR10*), P*_GAL1_* (*PDR11*), P*_GAL1_* (*PDR12*), P*_GAL1_* (*PDR15*), P*_GAL1_* (*PDR18*), P*_GAL1_* (*SNQ2*), P*_GAL1_* (*YOR1*), P*_GAL1_* (*AUS1*), P*_GAL1_* (*STE6*), P*_GAL1_* (*YOL075C*), P*_GAL1_* (*FZO1*), and P*_GAL1_* (*MGM1*).

**Additional Methods**

*Promoter replacement plasmids construction*

pBX-*PDR5* was taken as an example for detailed construction procedure of the promoter replacement plasmids (Fig. S1A). Amplified 3 fragments *GAL1* promoter, PDR5 F (487 bp upstream of the *PDR5* promoter) and PDR5 R (600 bp downstream of the *PDR5* promoter) from yeast FY1679-01B genomic DNA, and fused together by overlap extension PCR to generate the *GAL1* promoter-PDR5 R-PDR5 F structure. Restriction site *Bam*HI was introduced between PDR5 F and PDR5 R. Selection marker (*URA3* and *KanMX*) flanked by *loxp* sites were obtained from pUMRI-21 by double digestion (*Puv*II and *Bss*HII). The two segments were ligated to assemble the plasmid pBX-PDR5 by fusion cloning. A series of promoter replacement plasmids could be constructed according to this scheme.

*iTRAQ-based proteomic analysis*

Total protein from yeast cells was extracted using FOCUS™ Yeast Proteome kit (Sangon Biotech, China). The protein concentrations were measured by Quick Start Bradford Protein Assay (Bio-Rad) according to Bradford method and used for further treatment. For iTRAQ labeling, take 120 μg of each protein sample and make up the volume to 100 μL with lysis buffer, and subsequently digested with 3 μL of trypsin (1 μg/μL) and 500 μL of 50 mM TEAB buffer overnight at 37 °C. After the digested sample was desalted, add 20 μL of 1 M TEAB buffer to reconstitute, then add iTRAQ labeling reagent. Sample was shaking for 2 h at room temperature, and using 100 μL of 50 mM Tris-HCl (pH=8) to stop the reaction. The separated peptides were analyzed by Q Exactive HF mass spectrometer (Thermo Fisher), with ion source of Nanospray Flex^TM^ (ESI). The resulting spectra from each fraction were searched separately against *Saccharomyces cerevisiae* database by Proteome Discoverer 2.2. iTRAQ 8-plex is used for iTRAQ quantification. Statistical analysis of protein quantitative results by Mann-Whitney test to compare proteins with significant quantitative differences between the experimental group and the control group (*P* < 0.05 and fold change ≥ 1.2) were defined as differentially expressed proteins.

**References**

Verwaal, R., Wang, J., Meijnen, J.P., Visser, H., Sandmann, G., van den Berg, J.A., van Ooyen, A.J., 2007. High-level production of beta-carotene in *Saccharomyces cerevisiae* by successive transformation with carotenogenic genes from *Xanthophyllomyces dendrorhous*. Appl. Environ. Microbiol. 73, 4342-4350.

Lv, X., Wang, F., Zhou, P., Ye, L., Xie, W., Xu, H., Yu, H., 2016. Dual regulation of cytoplasmic and mitochondrial acetyl-CoA utilization for improved isoprene production in *Saccharomyces cerevisiae*. Nat. Commun. 7, 12851.
